# Stochasticity in the enterococcal sex pheromone response revealed by quantitative analysis of transcription in single cells

**DOI:** 10.1371/journal.pgen.1006878

**Published:** 2017-07-03

**Authors:** Rebecca J. Breuer, Arpan Bandyopadhyay, Sofie A. O’Brien, Aaron M. T. Barnes, Ryan C. Hunter, Wei-Shou Hu, Gary M. Dunny

**Affiliations:** 1 Department of Microbiology and Immunology, University of Minnesota, Minneapolis, Minnesota, United States of America; 2 Department of Chemical Engineering and Materials Science, University of Minnesota, Minneapolis, Minnesota, United States of America; 3 Department of Laboratory Medicine and Pathology, University of Minnesota Medical School, Minneapolis, Minnesota, United States of America; The University of Texas Health Science Center at Houston, UNITED STATES

## Abstract

In *Enterococcus faecalis*, sex pheromone-mediated transfer of antibiotic resistance plasmids can occur under unfavorable conditions, for example, when inducing pheromone concentrations are low and inhibiting pheromone concentrations are high. To better understand this paradox, we adapted fluorescence *in situ* hybridization chain reaction (HCR) methodology for simultaneous quantification of multiple *E*. *faecalis* transcripts at the single cell level. We present direct evidence for variability in the minimum period, maximum response level, and duration of response of individual cells to a specific inducing condition. Tracking of induction patterns of single cells temporally using a fluorescent reporter supported HCR findings. It also revealed subpopulations of rapid responders, even under low inducing pheromone concentrations where the overall response of the entire population was slow. The strong, rapid induction of small numbers of cells in cultures exposed to low pheromone concentrations is in agreement with predictions of a stochastic model of the enterococcal pheromone response. The previously documented complex regulatory circuitry controlling the pheromone response likely contributes to stochastic variation in this system. In addition to increasing our basic understanding of the biology of a horizontal gene transfer system regulated by cell-cell signaling, demonstration of the stochastic nature of the pheromone response also impacts any future efforts to develop therapeutic agents targeting the system. Quantitative single cell analysis using HCR also has great potential to elucidate important bacterial regulatory mechanisms not previously amenable to study at the single cell level, and to accelerate the pace of functional genomic studies.

## Author summary

Within a given niche, expression levels of individual cells (and resulting functional behaviors) may differ substantially from the mean of the population due to stochasticity or microenvironment heterogeneity. Quantification of bacterial gene expression at the single cell level provides a more informative picture of microbial communities. In *Enterococcus faecalis*, intercellular communication via peptide pheromones controls conjugation-mediated transfer of antibiotic resistance, adaptation to stress, and formation of biofilms. Population-level studies have shown that induction of conjugation by peptides is tightly controlled, but the extent of single cell variation in the process has not been explored. We analyzed induction in single cells by direct transcript labeling and GFP reporter expression and show that the response is heterogeneous and stochastic. Mathematical simulations modeled the components of the system and predicted this response. Importantly we show that conjugation can occur in response to low inducer peptide concentrations and in the presence of high inhibitory peptide concentrations. In both of these cases, some cells respond at a similar early time. Stochasticity in the response could explain the occurrence of induction and conjugation in these scenarios that might seem to disfavor plasmid transfer and permit plasmid dissemination while minimizing fitness costs of induction to donor populations.

## Introduction

**Fig 1:**
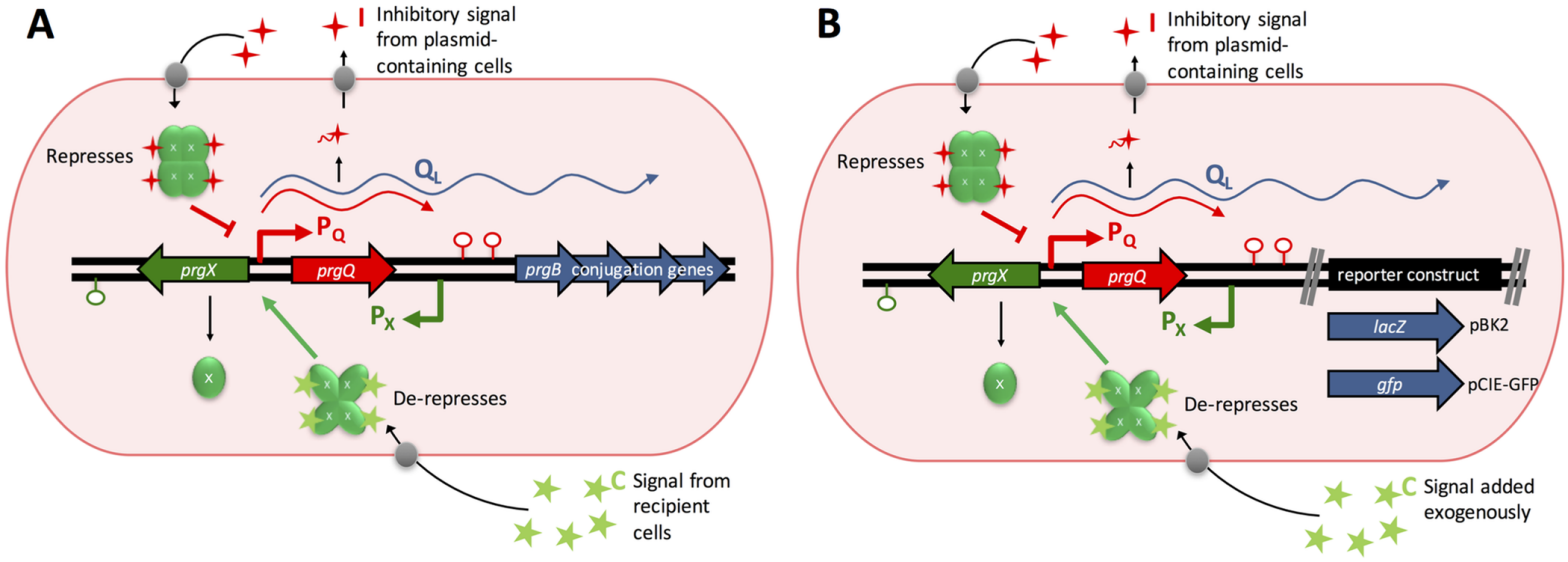
Model of induction of the pCF10 conjugative plasmid and reporter systems. **(A)** The Q_L_ transcript from the pCF10 plasmid encodes the proteins that mediate conjugation and is induced upon signaling by ***C*** pheromone (green stars) from potential recipient cells. The ***I*** inhibitory peptide (red stars) counteracts ***C*** and is produced by plasmid-containing cells from a short transcript from the P_Q_ promoter (red). PrgX complexes (green circles) repress the P_Q_ promoter whereby PrgX-***C*** complexes allow induction of Q_L_ transcription and PrgX or PrgX-***I*** complexes inhibit transcription of Q_L_. **(B)** The pBK2 and pCIE-GFP reporter plasmid constructs have the same P_Q_/ Q_L_ regulatory region as pCF10. However, either *lacZ* for pBK2 or *gfp* for pCIE-GFP have been inserted in place of the conjugation genes.

Enterococci are major contributors to the current antibiotic resistance crisis [1]. They are among the most common agents of antibiotic-resistant nosocomial infections, and their conjugative mobile genetic elements contribute to rapid intra- and intergenic horizontal transfer of resistance determinants [2–7]. Transfer of the tetracycline-resistance conjugative plasmid pCF10 between *E*. *faecalis* cells is controlled by two antagonistic signaling peptides (Fig 1A) [8]. A secreted pheromone cCF10 (***C;*** sequence LVTLVFV, originally termed “clumping-inducing” since it induces formation of visible cell aggregates) is produced by plasmid-free recipients and a secreted inhibitor peptide iCF10 (***I***; sequence AITLIFI) is encoded by the *prgQ* gene in the pCF10 plasmid [9–12]. In the absence of ***C***, the *prgQ* promoter (P_Q_) is repressed and basal transcription from the P_Q_ promoter terminates approximately 400 nt from the transcription start site at IRS1, (an inverted repeat sequence and terminator), resulting in a “short Q” (*Q*_*S*_) RNA. Induction of conjugation occurs when ***C*** is imported into plasmid carrying cells, where it binds to the master transcription regulator PrgX [13] and prevents repression of transcription from P_Q_ by PrgX. Increased levels of *prgQ* RNAs override counter-transcript-mediated attenuation at IRS1 to produce “long Q” transcripts (*Q*_*L*_) that can extend through the entire *prgQ* operon (Fig 1A and S1 Fig) [8,14]. *Q*_*L*_ RNAs are virtually undetectable in uninduced cells, but upon exposure to ***C*** they increase in a dose-dependent fashion [15,16] and result in expression of downstream conjugation genes. Induction of the *prgQ* operon increases the production of the inhibitor peptide ***I*** which competes with ***C*** for binding to PrgX; PrgX-***I*** complexes increase PrgX repression of P_Q_ (Fig 1A and 1B) [12,17,18]. While the basal levels of ***I*** produced from *Q*_*S*_ transcripts help prevent spurious induction in the absence of recipients, the increase in ***I*** following induction is essential for the rapid shut-down of the response [16]. The identification of multiple layers of positive and negative feedback loops operating in the pCF10 system and quantitative analysis of *prgQ* expression in populations of donor cells suggested that the system could function as a bistable switch [19,20].

Conjugative transfer of pCF10 in *E*. *faecalis* is population composition dependent. At low donor densities relative to recipients, the conjugation operon is induced at high levels and results in high conjugation frequencies. In contrast, high donor densities relative to recipients result in decreased induction of the conjugation operon and reduced conjugation frequencies [16]. This calibrated response by the donor population likely increases their fitness by reducing energy expenditure and potential deleterious effects of induction of conjugation on donor viability [21,22] when there is already a large population of donors carrying the plasmid. However, a reduced response to induction limits the opportunity for plasmid transfer to new recipients, which may be beneficial to both the plasmid and the new host. Hence, we hypothesized that the stochasticity in the enterococcal pheromone response allows for induction in a sub-population of donors even when the pheromone concentrations are insufficient to induce high levels of conjugation. Stochasticity will be reflected in the heterogeneity of the donor response to induction by ***C*** and will lead to a small number of cells responding to a very low concentration of ***C*** that is below the threshold for induction for the majority of cells.

Many past studies have characterized the pheromone response at the population level and revealed the dynamics of the response [8], but these studies were unable to detect heterogeneity of induced expression in individual cells that could have functional relevance [23]. In this study, we exposed populations of cells to pheromones and examined the induced response within single cells using direct transcript labeling and a fluorescent reporter system. These experimental methods, combined with mathematical modeling, demonstrated that the response is stochastic and heterogeneous. The stochastic nature of pheromone induction may directly account for the heterogeneity in the response that was observed and the occurrence of conjugation in the presence of low inducing pheromone concentrations and high inhibiting pheromone concentrations.

## Results

### Conjugation occurs when inducing pheromone concentrations are low and inhibiting pheromone concentrations are high

**Fig 2:**
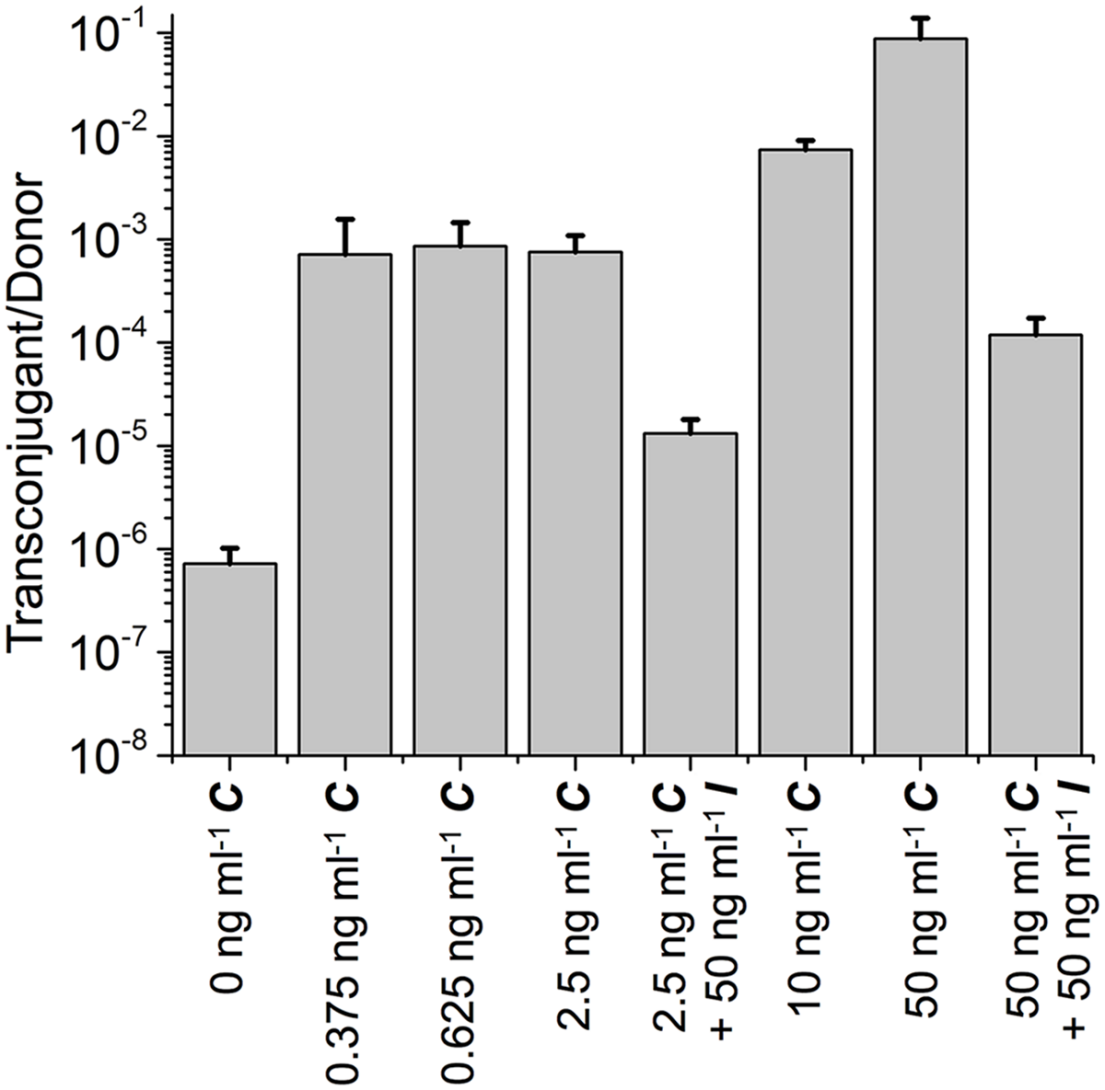
Conjugation occurs when inducing pheromone concentrations are low and inhibiting pheromone concentrations are high. *E*. *faecalis* cells carrying pCF10 were exposed to various concentrations of ***C*** and ***I*** as indicated on the horizontal axis. After 30 minutes, cells were mixed with plasmid-free recipients and allowed to mate during a 10 minute incubation as described in the Methods. After mating, cultures were plated on selective media to enumerate donors, recipients, and transconjugants. The y axis shows the number of transconjugants per donor and error bars represent standard deviation of 3 replicates.

A unique feature of cell-cell signaling in the enterococcal sex pheromone systems is the involvement of two antagonistic peptide signals, the chromosomally-encoded plasmid transfer inducing peptide ***C*** (mate-sensing signal), and the plasmid-encoded transfer inhibiting peptide ***I*** (self-sensing signal). We have shown that extracellular levels of ***I*** produced by uninduced donor cultures accumulate in a density-dependent fashion, functioning as a classic quorum sensing signal of donor population density [12]. ***C***-mediated induction is drastically reduced at high donor density, as determined by quantitative analysis of transcription in donor cultures. However, the notion that ***I*** can completely shut down induction is called into question by experiments looking at the conjugation efficiency of donor cultures exposed to various concentrations of ***C*** and high concentrations of ***I*** (Fig 2). In this experiment, donor cells were incubated with synthetic peptides for 30 minutes and then mixed with recipients for 10 minutes prior to plating on medium selective for transconjugants. This short mating time is only sufficient to allow the donors induced during the pre-incubation to undergo one round of conjugation. Under these very stringent mating conditions, fewer than 1 transconjugant per 10^6^ donors was produced when no exogenous peptides were added. At the other extreme, the frequency rose to nearly 10,000- fold (to nearly 1 transconjugant per 10 donors) when the donors were pre-exposed to a saturating concentration of 50 ng ml^-1^ ***C***. Exposure to intermediate ***C*** concentrations in the 0.3 to 2.5 ng ml^-1^ range (more closely resembling the levels of ***C*** naturally produced by recipient cells [12]) resulted in a jump to levels of transfer about 1,000-fold above uninduced cells, but well below the maximum. Most striking was the observation that induction with 2.5 ng ml^-1^ ***C*** in the presence of a great excess of ***I*** (50 ng ml^-1^) still increased transfer by a factor of 10. These data suggest that a very small number of donors may be induced to transfer under conditions where the vast majority of the population is uninduced. Confirmation that these results reflected a variable transcriptional response among this small subpopulation required analysis of the pheromone response at the single cell level.

### Pheromone induced and constitutive transcripts of *E*. *faecalis* can be visualized by fluorescence *in situ* hybridization chain reaction (HCR)

**Fig 3:**
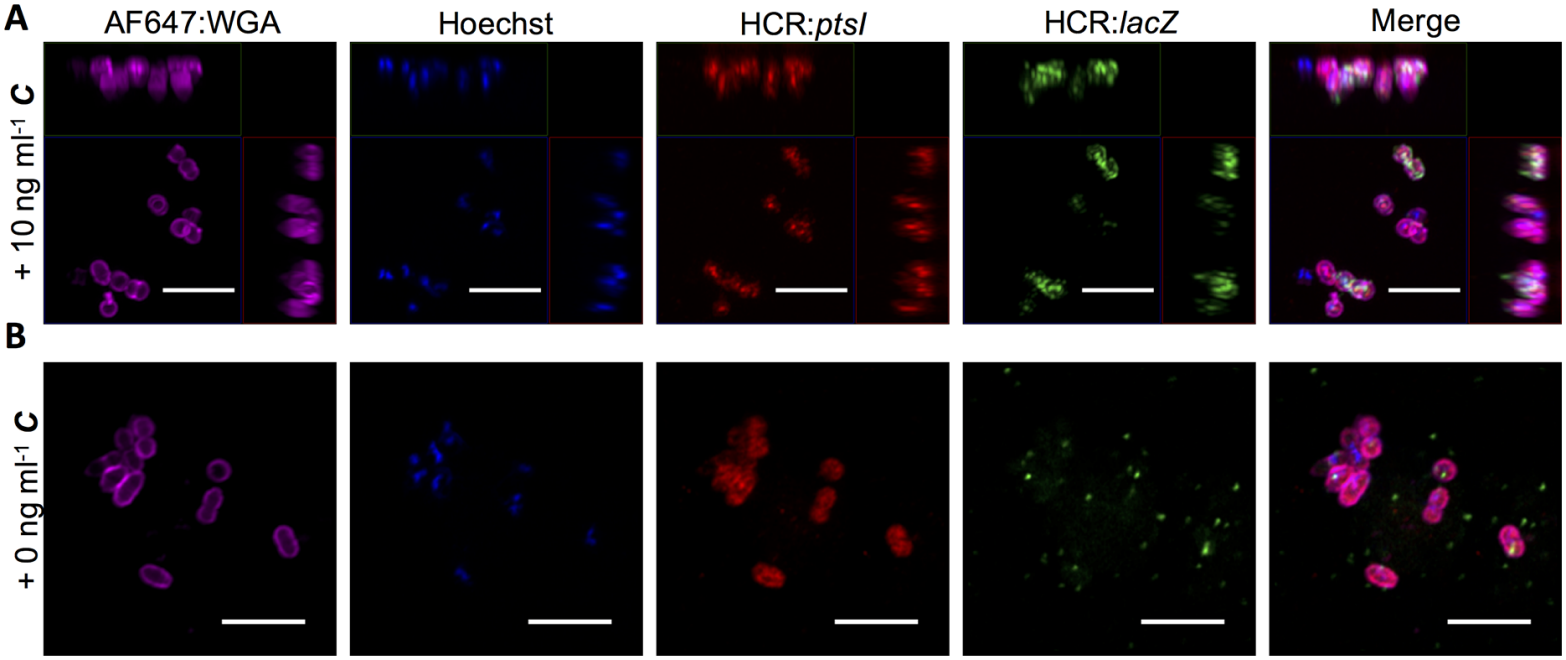
Visualization of pheromone induced and constitutive transcripts by fluorescence *in situ* hybridization chain reaction (HCR). Fluorescence images demonstrating simultaneous labeling of multiple transcripts in *E*. *faecalis* cells by fluorescence *in situ* hybridization chain reaction (HCR). Purple, *E*. *faecalis* cell envelope labeled with Alexa Fluor 647: wheat germ agglutinin (AF647: WGA) conjugate highlighting the outsides of individual cells. Blue, Hoechst 33342 DNA label also highlighting individual cells. Red, HCR labeled *ptsI* transcripts (Alexa Fluor 546). Green, HCR labeled *lacZ* transcripts (Alexa Fluor 488). **(A)** *E*. *faecalis* cells containing pBK2 30 minutes after addition of 10 ng ml^-1^ ***C***. Images are maximum intensity projections of Airyscan stacks and show z-axis projections. **(B)** *E*. *faecalis* cells containing pBK2 without addition of ***C***. Images are a single z-plane of an Airyscan processed image. The punctate green HCR *lacZ* signal observed without addition of ***C*** is weak and much less intense than the signal observed after addition of ***C***. This signal is visible in this figure due to intentional over exposure and the Min/Max brightness and contrast adjustment. Notably, these puncta are generally localized outside of cells and appear different than true signal observed with addition of ***C*** or the red HCR *ptsI* signal. See S4 Fig for further documentation. Scale bars, 5 μm.

Fluorescence *in situ* hybridization chain reaction (HCR) was recently developed to detect specific transcripts in a range of eukaryotic organisms [24] and in microbial symbionts of termites and the bobtail squid [25,26]. We adapted the technology to quantitatively analyze the dynamics and variability in the levels of pheromone-inducible transcripts of *E*. *faecalis* at the single cell level (S2 Fig). Initially, we used an *E*. *faecalis* strain carrying pBK2, which contains a pheromone-inducible *lacZ* reporter for Q_L_ expression, along with the native *prgX* and *prgQ* genes and promoters in their native configurations (Fig 1B). We used HCR probes against *lacZ* mRNA (paired with HCR amplifiers) to assay transcript levels (S1 Table). The *lacZ* reporter allowed for rapid independent confirmation of the induction state of cultures. Simultaneous labeling of transcripts from the constitutively expressed chromosomal gene *ptsI* was also performed by HCR (S1 Table). The fluorescent HCR signal of *lacZ* mRNA in cells induced for 30 min with a high concentration of ***C*** was very strong in most cells (Fig 3A) compared to that of uninduced cells (Fig 3B). Virtually all cells, both induced and uninduced, labeled strongly with cell envelope and nucleoid stains, as well as with the HCR probes for *ptsI* mRNA, suggesting that our labeling protocol (S3 Fig) effectively permeabilized cells to the HCR probes without lysis. The rare green fluorescent particles observed in preparations of uninduced cells were of low intensity and generally localized outside of cells (Fig 3B and S4 Fig). These results indicated the feasibility of using HCR for more detailed quantitative single-cell analysis of multiple transcripts in *E*. *faecalis*.

### HCR and GFP reporter analysis of the induction response demonstrate heterogeneity within responding populations over time

**Fig 4:**
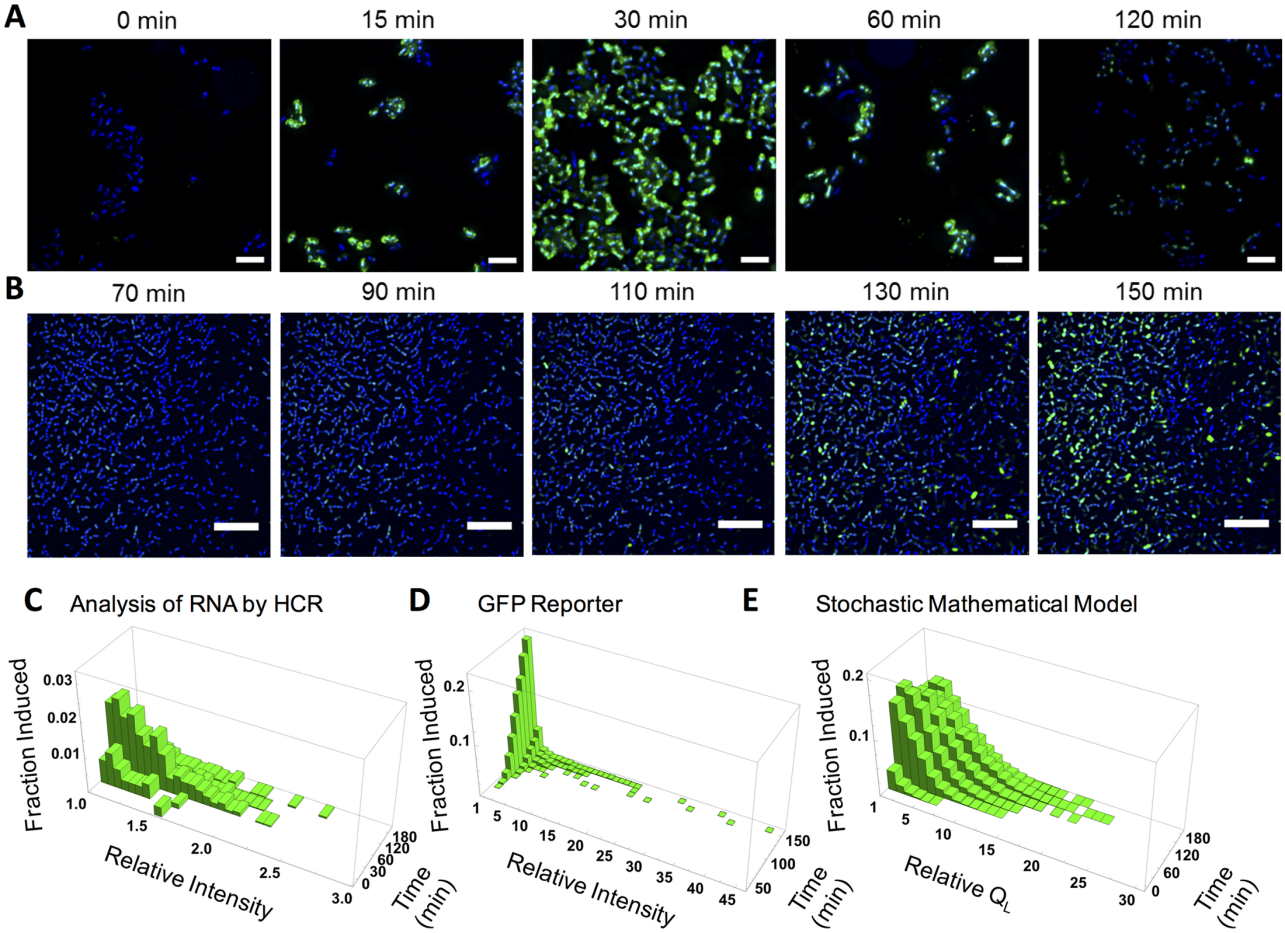
Analysis of the induction response using either HCR or a GFP reporter demonstrates heterogeneity within responding populations over time. **(A)** Time course showing *lacZ* expression in *E*. *faecalis* upon induction with 10 ng ml^-1^ ***C***. Times (left to right): 0, 15, 30, 60, and 120 minutes after ***C*** addition. Green, HCR labeled *lacZ* transcripts (pseudo colored Alexa Fluor 546). Blue, Hoechst 33342 DNA label highlighting individual cells. **(B)** Time course of GFP expression in *E*. *faecalis* upon induction with 5 ng ml^-1^ ***C***. Times (left to right): 70, 90, 110, 130, and 150 minutes after ***C*** addition. Green, GFP. Blue, Hoechst 33342. **(C)**, **(D)**, and **(E)**: 3D distributions reflecting the fraction of cells induced over time as measured by HCR, GFP expression, or predicted by the stochastic model respectively. Relative intensity or Q_L_ of induced cells was normalized to the threshold value and reflects varied levels of induction. **(C)**, Induction of *lacZ* RNA over time from pBK2 plasmid upon addition of 5 ng ml^-1^ ***C*** in a ***C***^***-***^ host as shown by relative HCR fluorescent intensity per cell. **(D)** Induction of fluorescent GFP over time from pCIE-GFP plasmid upon addition of 5 ng ml^-1^ ***C*** in a ***C***^***-***^ host as shown by relative fluorescent intensity per cell. **(E)** 3D distributions of the induced expression of the Q_L_ transcript upon addition of 5 ng ml^-1^ ***C*** in a population of cells over time simulated using the stochastic mathematical model. The fraction induced reflects the proportion of cells with the depicted levels of Q_L_ out of the total cell population. Scale bars, 3.9 μm **(A)** and 20 μm **(B)**.

Cells carrying a pheromone-inducible *lacZ* reporter (the pBK2 plasmid) (Fig 1B) were induced with a high concentration of ***C***, fixed at a range of time points, and assayed for *lacZ* expression by HCR (Fig 4A). Notably, *lacZ* mRNA was detected within 15 minutes after ***C*** addition, and reached maximum levels within 30 to 60 minutes. By 120 minutes, the number of cells expressing *lacZ* was significantly reduced (Fig 4A). These results reflect the temporal response that has been observed in the past using population- based methods such as qRT-PCR, RNAseq, and microarray [16,27].The induction process was also examined using an inducible GFP reporter (pCIE-GFP; Fig 1B), and the onset of induction was similarly observed. GFP fluorescence was seen approximately 60 min after ***C*** addition (Fig 4B and S1 Movie). The delayed response compared to HCR is likely caused by the time required for protein folding after translation. Due to the inherent stability of the GFP protein, a decrease in fluorescence was not observed even though the analogously induced *lacZ* transcript level had decreased as seen with HCR. Using the GFP construct further allowed for tracking gene expression in individual cells over time using time-lapse microscopy which is not possible using HCR as it requires fixed cells. Hence, the two methods were complimentary in revealing the population dynamics of induction by ***C***. Both methods showed heterogeneity in that some cells became induced earlier than others and that the signal intensities were not the same in every induced cell.

We quantified the fluorescent intensity of HCR or GFP signals of individual cells at various time points following ***C*** addition (Fig 4C and 4D and S5, S6, S7 and S8 Figs). An uninduced cell population (0 ng ml^-1^ ***C***) was used to establish a strict threshold which was then used to categorize cells as induced or uninduced at different time points (Fig 4C and 4D). Among the uninduced population, a very small number of cells (0.3%) had a high HCR fluorescence intensity, likely due to artifacts of the labeling method. After removing those outliers, the maximum intensity of the uninduced population was used as the threshold. The intensity of induced cells was normalized to the threshold intensity. Using a strict threshold helped distinguish genuine response from noise, but may also have resulted in an elevated number of false negative cells. With both GFP and HCR, the signal intensity of individual cells at any given time point varied widely (Fig 4C and 4D). As time progressed the fraction of cells which were induced increased.

### Stochastic modeling of the induction response predicts heterogeneity within responding populations over time

We had previously developed an ordinary differential equation (ODE)-based mathematical model describing the induction process [16]. In light of new knowledge on the oligomeric states of apo-PrgX and PrgX/peptide complexes [28], availability of kinetic parameters, and a greater mechanistic understanding of its interactions with DNA, the model was refined (S1 Fig). A stochastic model based on our updated model was developed using Hy3S [29] which consists of 15 species and 38 biochemical reactions and interactions involved in the induction of pCF10 (S1 Text and S2 Table). The Q_L_ transcript level in response to ***C*** was simulated in 10,000 individual cells (Fig 4E). The simulated time dynamics of Q_L_ behaved similarly to experimentally observed HCR and GFP profiles. The fractions of induced cells increase over time and after 150 minutes the population of highly induced cells shows a reduction indicating response shut-down.

### HCR analysis of reporter and pCF10 plasmids show similar dynamic induction responses to varied concentrations of *C*

**Fig 5:**
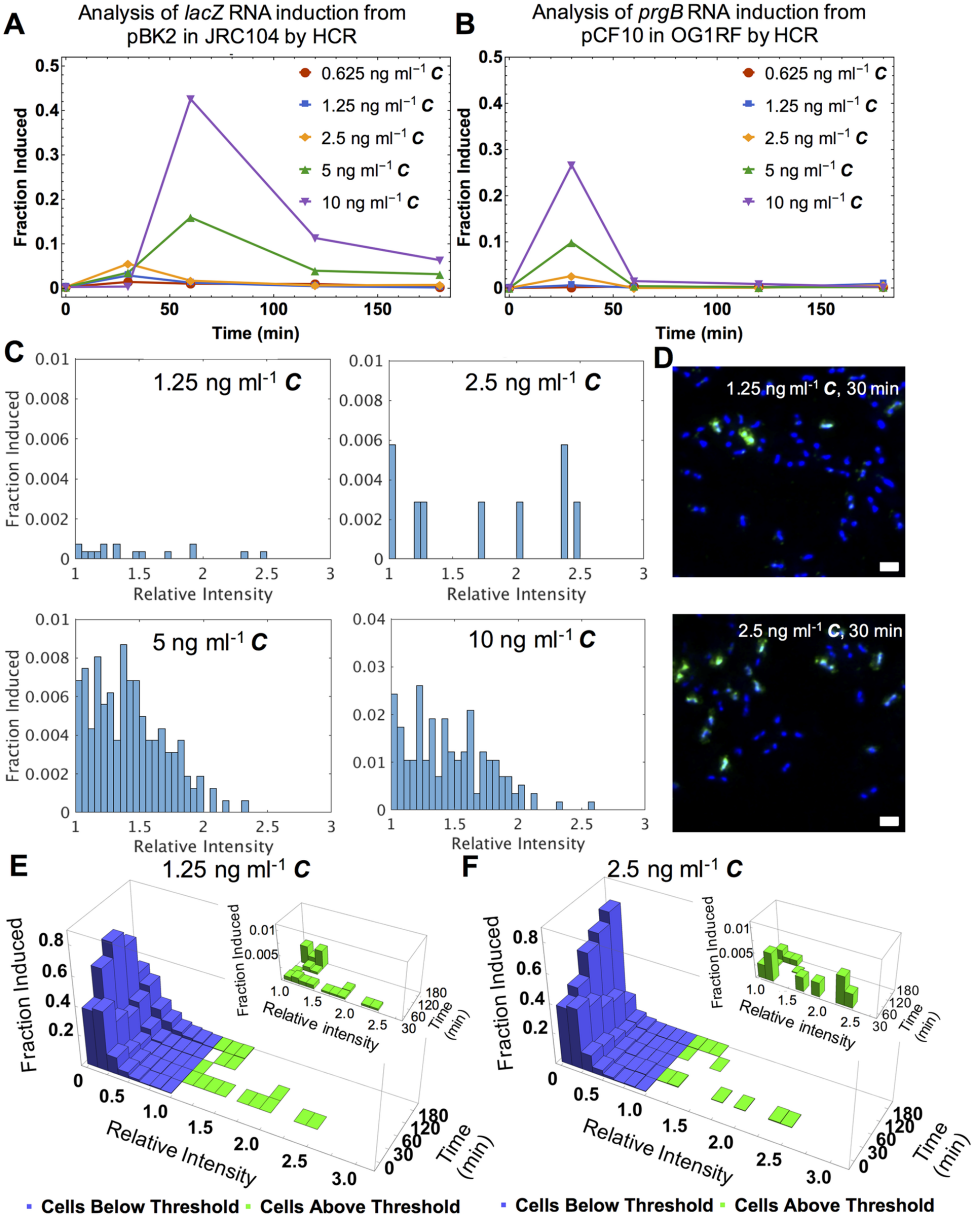
HCR analysis of reporter and pCF10 plasmids show similar dynamic induction responses to varied concentrations of *C*. **The frequency of induced cells at low concentrations of *C* is low, but highly-induced cells are present and these cells exhibit the same time course of induction as cells exposed to higher levels of *C*. (A)** and **(B)**: Fraction of cells with induced *lacZ* and *prgB* RNAs, expressed from pBK2 and pCF10 respectively, labeled by HCR over time. **(C)** Histograms show the fraction of cells with HCR labeled *prgB* expressed upon pCF10 induction in OG1RF 30 minutes after addition of varied concentrations of ***C***. Relative intensity of induced cells was normalized to the threshold value and reflects varied levels of induction. **(D)** Fluorescence images of *E*. *faecalis* with *prgB* transcripts labeled by HCR at 30 minutes after addition of 1.25 and 2.5 ng ml^-1^ ***C*.** Green, HCR labeled *prgB* transcripts. Blue, Hoechst 33342. Scale bars, 1.58 μm. **(E)** and **(F):** 3D distributions of the induced expression of the *prgB* transcript measured using HCR upon addition of 1.25 and 2.5 ng ml^-1^ ***C*** shown by relative HCR fluorescent intensity per cell over time. Relative intensity is normalized to the threshold value. Blue and green bars indicate the cells are below and above the threshold respectively. Inset shows induced cells with an expanded y axis.

We varied the concentrations of ***C*** from 0.625 ng ml^-1^ to 10 ng ml^-1^ and used HCR to examine the fraction of cells induced over time (Fig 5A). From past studies, we expected the population-averaged Q_L_ transcript level to increase to a higher level with increasing ***C*** and decrease rapidly after reaching a peak [19]. Using HCR we saw the fraction of cells induced increase with increasing ***C*** concentrations (Fig 5A). We further designed probes to the *prgB* transcript and directly probed the dynamics of the Q_L_ transcript of conjugation in cells harboring the wild type pCF10 plasmid (Figs 5B and 1A and S1 Table). The induction profile of *prgB* from pCF10 was similar in magnitude and time course to *lacZ* from pBK2 (Fig 5A and 5B). The faster increase and subsequent decrease in induction observed with pCF10 likely relates to increased import of the ***C*** and ***I*** peptides due to the presence of the pCF10 PrgZ peptide binding protein [30]. However, the overall similarity suggests that pBK2 contains the key regulatory components required for heterogeneous induction of pCF10. Additionally, flow cytometry analysis of the *lacZ* and *prgB* HCR fluorescence of our samples showed a similar induction profile (S9 Fig, S3 Table and S1 Methods).

### While the frequency of induced cells at low concentrations of *C* is low, highly -induced cells are still present under these conditions

The heterogeneous response to varying concentrations of ***C*** is not only reflected in the fraction of cells induced (i.e. having a fluorescence level above the threshold), but also in the transcript level among those induced. Variation in transcript level can be seen in the plot of HCR signal intensity distribution of the induced cells at 30 minutes post-induction (Fig 5C). Using qRT-PCR measurement (a population averaging assay), we had previously reported induction at 1 ng ml^-1^ ***C*** [16]. Using HCR we followed induction on the single cell level and saw that even at ***C*** concentrations as low as 1.25 ng ml^-1^ a few cells were induced to levels seen in populations exposed to higher ***C*** concentrations (Fig 5C). Samples of these rare, highly induced cells were readily visualized (Fig 5D) and this small fraction of highly induced cells at 1.25 and 2.5 ng ml^-1^ of ***C*** subsequently subsided over a similar time duration to that observed with higher ***C*** concentrations (Fig 5E and 5F). The observation of a few highly induced cells at low pheromone concentrations where the vast majority of cells remained uninduced suggests that stochasticity is at play in this system.

### Distribution of induction times for *E*. *faecalis* cells exposed to exogenous *C* and *I* show early responders for low *C* concentrations by both the GFP reporter and model analysis in the presence and absence of *I*

**Fig 6:**
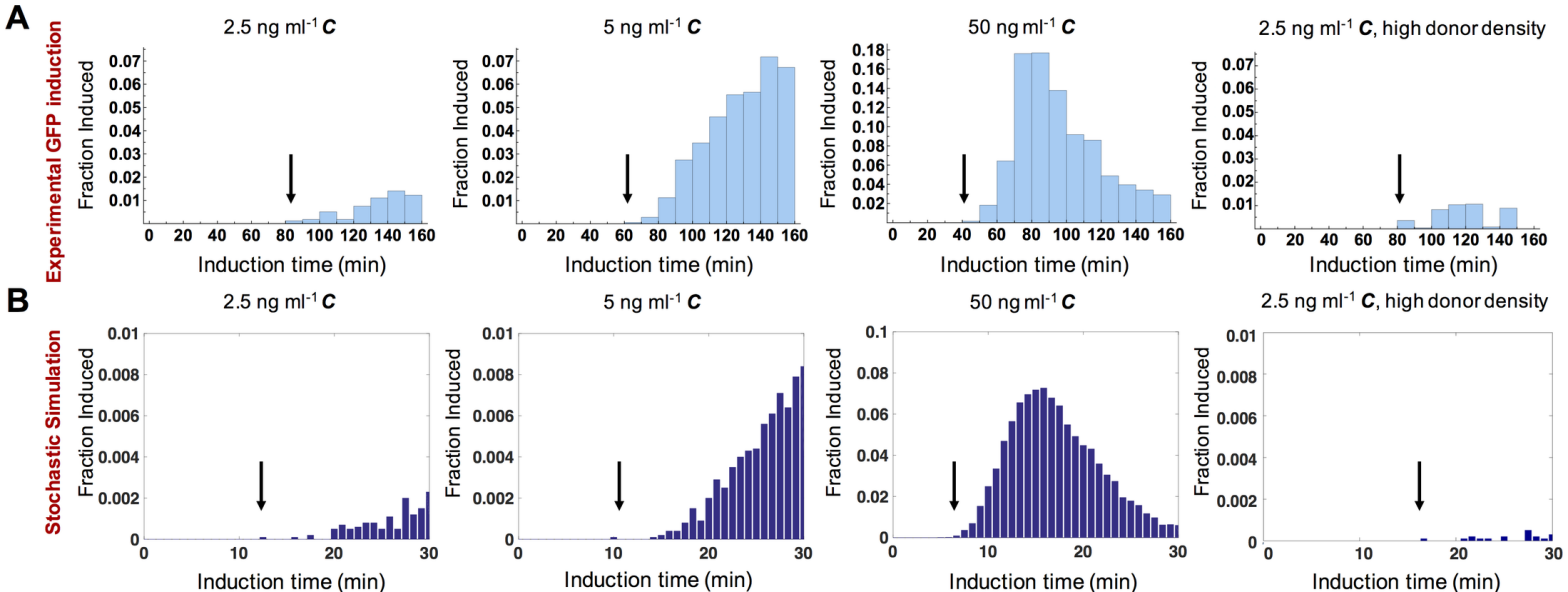
Distribution of induction times for *E*. *faecalis* cells exposed to exogenous *C* and *I* show early responders in tested conditions by GFP and model analysis. **(A)** Experimentally obtained time distributions showing the fraction of cells crossing the GFP induction threshold. The experiment in the far right panel mimics high donor density by the presence of a high concentration of ***I*** (50 ng ml^-1^). **(B)** Predicted time distributions of the fraction of cells crossing the induction threshold determined by stochastic simulations. (**A**) and (**B**): Induction time refers to the time window during which an individual cell became induced and fraction induced refers to the fraction of cells that became induced in that time window. Black arrows indicate the time at which the first cell within the observed population became induced.

We next tracked the induction at different ***C*** concentrations using the GFP reporter and time lapse microscopy. The time point that each cell became induced (the first time point at which GFP fluorescence was observed) after exposure to ***C*** was determined and plotted (Fig 6A). At a high inducer concentration of 50 ng ml^-1^, the earliest induction occurred around 40 minutes after ***C*** addition, while the median induction time was around 90 minutes. At later time points, when the majority of cells had been induced, the frequency of newly induced cells decreased. At lower ***C*** concentrations of 2.5 and 5 ng ml^-1^, the median time to initial induction shifted to longer time points, and even at 160 minutes cells continued to be induced at a relatively high rate. Importantly, even though the population median induction time was delayed in low versus high ***C*** concentrations, the time at which the first cells became induced was similar irrespective of ***C*** concentration (around 40–80 minutes as determined by GFP expression) (Fig 6A). When we used the stochastic model to simulate the response of cells to varying concentrations of ***C***, the model (Fig 6B) predicted the behavior observed experimentally using GFP (Fig 6A). Further, in the presence of high concentrations of ***I*** (50 ng ml^-1^), reflecting the conditions that occur at high donor densities, low ***C*** concentrations (2.5 ng ml^-1^) still result in early responding cells at a low frequency (Fig 6A and 6B).

## Discussion

It has been shown previously that the response of *E*. *faecalis* to induction by ***C*** signaling (required for transfer of the pCF10 conjugative plasmid) is concentration dependent, robust, and rapid on the population level (reviewed in [8]**)**. However, at low recipient and high donor cell density (and thus low ***C*** concentrations and high ***I*** levels), low but statistically significant levels of conjugation were observed (Fig 2). To gain insights into the induction response in these conditions, we performed single cell analysis of the induction response using multiple complementary methods (Figs 4, 5 and 6). The results obtained from HCR transcript labeling and reporter experiments indicate that isogenic populations of donor cells exposed to varied concentrations of ***C*** respond heterogeneously. Model simulation supports that the phenomenon of heterogeneity and early induction can be attributed to the stochasticity in the induction system.

### Significance of a stochastic induction response

Stochasticity in the induction response resulted in early induction of some donor cells, even under low ***C*** conditions unfavorable to induction (Fig 6), and allowed a small subpopulation of cells to undergo conjugation while the vast majority of cells remained in an uninduced state (Fig 2). The occurrence of rare conjugation events in a bacterial community with a high density of plasmid-containing cells may help spread the plasmid to recipient cells which may carry other critical traits. We hypothesize that stochasticity in molecular interactions in the pheromone response circuit result in heterogeneity and stochasticity in the population response to induction.

### Possible sources of response stochasticity

At the single donor cell level, the concentrations of ***C*** and ***I*** are critical determinants of the induction state. With extracellular concentrations in the nM range, the intracellular levels of ***C*** and ***I*** are a few molecules per cell, as are PrgX complexes with ***C*** and ***I*** [12]. There are about 5 copies of the pCF10 plasmid per cell, and each copy contains a single functional operator target for PrgX regulation. Therefore, the intracellular levels of the critical molecular species controlling the pCF10 pheromone response are in the range of a small number of molecules per cell.

The induction of the conjugation system is driven by a series of molecular events (Fig 1) involving import of the peptides ***C*** and ***I***, their binding with PrgX to form PrgX-peptide tetramers, and their interaction with the operator site which regulates the access of RNA polymerase to the P_Q_ promoter [28]. The probability of each molecular event depends upon the concentrations of the molecular entities involved. The low intracellular levels of these entities can magnify stochastic behavior, causing individual cells to have different numbers of each of these molecules per cell and thus different outcomes. Even under unfavorable induction conditions of low ***C*** and high ***I***, the probability of a PrgX-***C*** complex displacing the repressing PrgX or PrgX-***I*** complexes from the operator (and leading to induction) is low but non-zero. Hence, stochasticity can lead to rare induction even under unfavorable conditions.

Since the intracellular concentrations of the peptides are one of the major sources of stochasticity, the rate of peptide import by the donor could significantly impact the induction and conjugation response. In the case of wild type donors, this process is mediated by the cooperative functions of the plasmid-encoded PrgZ pheromone binding protein and the chromosomal oligopeptide permease [30]. Thus, examination of peptide import at the single cell level could provide new insights into the induction process.

### Heterogeneity in the pheromone response and fitness

A heterogeneous pheromone response may benefit the bacterial community by balancing the benefits of dissemination of potentially beneficial plasmid genes with fitness costs due to expression of the large set of genes required for conjugative transfer [21,22]. Our results may contribute to an improved understanding of the evolutionary significance of stochastic variation in the enterococcal pheromone response and other microbial communication systems. In future work, it would be worthwhile to examine the transcription of additional genes in the distal segments of the long pheromone-inducible operon (Fig 1A) and quantify actual conjugation events at the individual cell level. Variability in the induction of downstream genes among the cells induced for expression of proximal conjugation genes would suggest the possibility that only some individuals in an “induced” population subset actually function in plasmid transfer. In this division-of-labor model, the remaining induced donors could contribute to cooperative behaviors such as formation of cell aggregates, and to the rapid shut down of the pheromone response by production of ***I***.

### Practical applications of this work

A better understanding of the stochastic behavior of this system may also benefit the development of novel therapeutic approaches for the prevention or treatment of opportunistic infections by multi-drug resistant enterococci. It might be possible to manipulate pheromone signaling to alter the population balance of commensal and resistant pathogenic strains as a therapeutic strategy [31]. However, stochastic variation affecting antibiotic resistance plasmid transfer should bring caution to such strategies. Any therapeutic agents that target bacteria responding to the normal peptide signals may affect the majority of the population, but fail to eradicate unresponsive bacteria, analogous to the function of persister cells in biofilm-associated antibiotic resistance [32].

Finally, with the ability to determine the transcript level of any gene using specific probes, HCR should facilitate quantitative analysis of the expression dynamics of many genes at the single cell level for many different microorganisms in addition to enterococci.

## Methods

### Bacterial strains, plasmids and inducer peptides

The strains and plasmids used are listed in S4 Table. All bacterial strains in this study were derived from *E*. *faecalis* strain OG1 [33] and the endogenous conjugative plasmid pCF10[34,35]. OG1RF is an OG1 derivative with rifampicin and fusidic acid resistance [34,36]. OG1Sp is an OG1 derivative with spectinomycin resistance [37]. JRC104 is an OG1RF derivative that does not produce the cCF10 peptide due to a nonsense point mutation in the cCF10 encoding *ccfA* gene [37]. pBK2 contains the pCF10 *prgX-Q* regulatory region with a *lacZ* fusion downstream of IRS1 [38], while pCIE-GFP was similarly constructed but with a *gfp* fusion [39].

### Growth conditions

*E*. *faecalis* strains were grown statically at 37°C in M9 medium containing 3 g l^-1^ yeast extract, 10 g l^-1^ casamino acids, 36 g l^-1^ glucose, 0.12 g l^-1^ MgSO_4_, and 0.011 g l^-1^ CaCl_2_. M9 medium was supplemented with 20 μg ml^-1^ chloramphenicol in overnight cultures of JRC104+pCIE-GFP to ensure maintenance of the plasmid. Antibiotic concentrations used in the mating experiment were 250 μg ml^-1^ for spectinomycin, 200 μg ml^-1^ for rifampicin, and 25 μg ml^-1^ for fusidic acid.

### Mating experiment

*E*. *faecalis* cultures of donor (OG1Sp+pCF10) and recipient cells (JRC104) were grown overnight at 37°C in 3 ml of M9 medium. Overnight cultures were centrifuged, washed twice with 1 ml KPBS containing 2 mM EDTA, and diluted 1:5 in fresh M9 medium. The cultures were incubated for 1 hour at 37°C and various concentrations of the ***C*** pheromone (cCF10) and ***I*** inhibitor (iCF10) were added to the donor culture followed by 30 minute incubation at 37°C. The donors and recipients were then mixed in 1:1 ratio and mating was carried out at 37°C for 10 minutes. Serial dilutions of these samples were plated on selective THB agar medium containing 30 g l^-1^ Bacto Todd Hewitt Broth (Becton, Dickinson and Company) and 15 g l^-1^ agar to enumerate the donors (spectinomycin and chloramphenicol resistant), recipients (rifampicin and fusidic acid resistant), and transconjugants (rifampicin, fusidic acid, and chloramphenicol resistant).

### Induction, cell harvest, and fixation for analysis by HCR

Overnight cultures were sub-cultured 1:10 in M9 medium and grown to early exponential phase (≈3 h to OD_600_ ≈ 1.2). Cultures were induced with between 0.625 ng ml^-1^ and 10 ng ml^-1^ cCF10 (***C***) peptide and cells were harvested at times 0 to 180 minutes after ***C*** addition. Fixation of cells began immediately upon harvest when they were mixed 1:1 with EM-grade 8% paraformaldehyde (PFA; 4% final concentration) and fixed for >20 h at 4°C. Following fixation, cells were isolated by centrifugation at 13,000×*g* for 5 minutes and subsequently resuspended in KPBS with trace RNaseOUT (Invitrogen).

### Fluorescence *in situ* hybridization chain reaction (HCR), cell staining, and mounting

Nucleic acid probes and hairpin amplifier sequences used in this study were obtained from Molecular Instruments (www.molecularinstruments.org), (S1 Table). To label transcripts (*ptsI*, *lacZ*, and *prgB*) in *E*. *faecalis*, established HCR methodology [24] was adapted to label 20 μl aliquots of cells in suspension at a time [25,26,40–43]. Briefly, cells were permeabilized, DNA probes were hybridized to transcripts of interest, fluorescent amplifier hairpins were hybridized to the bound probes, and subsequently cells were counterstained and mounted for microscopy. Between each re-suspension step, cells were pelleted by centrifugation at 13,000×*g* for 2 minutes and re-suspended in the subsequent buffer or reagent.

Our HCR protocol was as follows. For permeabilization, cells were suspended in 20 μl of permeabilization buffer containing 5 mg ml^-1^ lysozyme (Sigma-Aldrich), 0.1 M Tris/ HCl, 0.05 M EDTA, and trace RNaseOUT and incubated at 37°C for 3 h. After permeabilization, cells were suspended in 20 μl KPBS to wash and then prehybridized in 20 μl of the probe hybridization buffer (Molecular Instruments) for 30 minutes at 45°C. Next, cells were suspended in 20 μl of probe hybridization buffer preheated to 45°C and containing 2 nM of each probe to the transcripts of interest (5 to 6 probes per transcript) then incubated at 45°C for >20 h. Following probe hybridization, cells were washed twice in 20 μl wash buffer (Molecular Instruments) for 30 minutes each at 45°C. Cells were then suspended in 20 μl amplification buffer (Molecular Instruments) for 30 minutes at room temperature for pre-amplification. Meanwhile required amplifier hairpins were heated in individual tubes to 95°C for 90 seconds in a thermocycler and then cooled to room temperature in a dark drawer for approximately 30 minutes. After pre-amplification, cells were suspended in 20 μl of amplification buffer containing 60 nM of each hairpin as appropriate and incubated in the dark at room temperature for >20 h. Probe sequences and amplifier details for each transcript of interest can be found in S1 Table. Following amplification, cells were washed twice in 20 μl 5× sodium chloride sodium citrate, Tween 20 (5× SSCT) (Molecular Instruments) in the dark for 30 minutes each at room temperature. To counterstain, cells were suspended in solutions of Hoechst 33342 nucleic acid stain (Thermo Fisher) and Alexa Fluor 647: wheat germ agglutinin (WGA) conjugate to label the cell envelope (Invitrogen). Cells were washed in KPBS, suspended in ddH_2_O, and then 10 μl of each suspension was applied to 22×22 mm No. 1.5 coverslips (Gold-Seal) to dry, and then mounted in hardening Prolong Diamond Antifade Mountant (Molecular Probes by Life Technologies). Mountant was allowed to harden for >48 h at 4°C before imaging.

### Microscopy and image processing for HCR

Images shown in Fig 3 were taken using a Zeiss Axio Observer.Z1 confocal microscope equipped with an LSM 800-based Airyscan super-resolution detector system (Zeiss). Confocal images were acquired through a 63×, 1.40- numerical aperture (NA) objective (Zeiss) in Airyscan mode. Images in Fig 3A were acquired as z stacks at 0.15- μm intervals, deconvolved through Airyscan processing, flattened using a maximum intensity projection with Ortho Display, and presented for publication as a Min/Max projection using Zen software (version 2.1, Zeiss). Images in Fig 3B were acquired in Airyscan mode at a single z plane, deconvolved, and similarly presented for publication as a Min/Max projection using Zen software. In this Min/Max projection, the Alexa Fluor 488 channel (corresponding to HCR labeled *lacZ* transcripts) appears to have some small bright puncta that appear markedly different from the HCR signal and rarely overlap with cells. While these puncta appear bright, their actual intensity is minimal in comparison to true HCR signal intensity as demonstrated in S4 Fig.

Images shown in Figs 4A and 5D were taken using a Nikon E-800 microscope equipped with a spinning disc BD CARV II confocal image adapter (BD Biosciences). A Cascade 1k EMCCD camera (Photometrics) was used to acquire images as wide-field z stacks at 0.2- μm intervals through a 100×, 1.45- NA objective (Nikon Instruments). Image z stacks were deconvolved using Huygens Professional software (version 4.5.0p8, Scientific Volume Imaging). The images shown here are cropped ImageJ (version 1.49m, NIH) maximum- intensity projections of the deconvolved z stacks with background subtracted using a rolling ball radius of 50.0 pixels.

Images that were used for image analysis (Figs 4C, 5A, 5B, 5C and 5E and S3, S5, S6, S7 and S8 Figs) were taken using an Olympus IX83- P2ZF inverted microscope equipped with an X-Cite 120LED light source (Excelitas Technologies) for fluorescent excitation. Emission filters were 409 nm, 506 nm, and 562 nm for the Hoechst 33342 stain, Alexa Fluor 488, and Alexa Fluor 546, respectively. A Hamamatsu C11440 Orca-Flash 4.0 CMOS camera was used to acquire images as wide-field z stacks at 0.24- μm intervals through a 60×, 1.42 NA objective (Olympus). Image z stacks were deconvolved using Huygens Professional software, then flattened using a maximum- intensity projection, and subjected to background subtraction using a rolling ball radius of 50.0 pixels in ImageJ (version 1.49m, NIH) before image analysis in Matlab (version 2015b, Mathworks) as described below.

### Image analysis for HCR

In brief, the blue fluorescence channel (the reference channel corresponding to Hoechst nucleic acid labeling) was used as a proxy to define the pixel locations of individual cells and co-localized fluorescent overlap from HCR labeled transcripts was quantified (S5 and S6 Figs). The overall image analysis scheme can be found in the Supplementary Information. Images corresponding to each fluorescent channel (that had been deconvolved and then flattened by maximum intensity projection and subjected to background subtraction) were imported into Matlab (version 2015b, Mathworks) in 16 bit TIFF format. To identify cell positions, the blue fluorescence (reference channel) images were binarized using Otsu’s method [44] for thresholding. The internal Regionprops function was used to identify and characterize the sizes and pixel locations of objects in the image. From there, objects less than 3 pixels or greater than 30 pixels were filtered out from the analysis using find and ismember functions. S6 Fig shows that this filtering strategy was effective in identifying cells. Objects between 3 and 30 pixels in size were analyzed as cells in subsequent analysis. The PixelList property from the Regionprops function (called on the blue reference channel) was used to define the pixels corresponding to each cell. Using these coordinates, the HCR intensity value corresponding to each cell was calculated by taking the mean intensity of the pixels corresponding to each cell. Graphs were created using Matlab and Mathematica (version 11.0.1.0, Wolfram).

### Induction and microscopy for GFP analysis

100 μl of an overnight culture of JRC104+pCIE-GFP diluted 1:3 in M9 medium was added to a poly-D-Lysine coated glass bottom 96 well plate (No. 1.5, MatTek Corporation). The cells were incubated at 37°C for 1 h, and then washed with KPBS to remove loose cells. M9 media containing Hoechst 33342 and cCF10 at concentrations of 0, 2.5, 5, or 50 ng ml^-1^ was then added to the cells. For experiments involving iCF10, 50 ng ml^-1^ iCF10 was added to the cells in addition to cCF10. Cells were imaged on a Zeiss Axio Observer Z1 inverted confocal microscope (Zeiss Zen 2.0) using the 405 nm and 488 nm lasers for excitation of Hoechst and GFP, respectively. A 63×, 1.4 NA objective was used. Images were taken every 10 minutes from 40 to 150 minutes after addition of cCF10. Two fields of view were imaged for each cCF10 concentration and ten z-stacks were taken at 0.26 μm intervals for each image.

### Image processing and analysis for GFP images

Images were imported into ImageJ and background subtracted using a radius of 4 pixels (ImageJ version 1.50a, NIH). For the blue fluorescence channel (the reference channel corresponding to Hoechst fluorescence), a maximum intensity z-projection was used to flatten the z-stacks (ImageJ). For the GFP fluorescence channel, the Sum Slices z-projection was used to flatten the z-stacks (ImageJ). These processed images were then imported into Mathematica for further analysis. To identify cell positions, the blue fluorescence (reference channel) images from the first time point were binarized and then distance transform and maximum detection functions were applied to create cell location markers. Then, using the markers previously found as positional information, a watershed transformation was applied to the binarized images and the SelectComponents function was used to identify any object found by the watershed algorithm between 3 pixels and 2.5x the mean object size. The ComponentsMeasurements function was then used to find the centroid coordinates and the equivalent disk radius of all identified cells. Using these coordinates as starting points, the algorithm ImageFeatureTrack was applied to all images within the time series to track the cells through different images and counteract microscopic drift. Any cells which were unable to be tracked by the algorithm through all time points were removed from further analysis. To identify the GFP fluorescence intensity for each cell over time, the centroid location and equivalent disk radius for each cell in each image were used to create a mask. When applied to the GFP fluorescence images, the mask set all pixels equal to zero except for the area surrounding a specific cell. The GFP intensity of the cell at that time was then calculated by taking the mean intensity of the non-zero pixels. This process was repeated for each cell in each image at each time point. Using this method, the GFP intensity for each cell over time was determined. Graphs were created using Mathematica. An overview of this analysis strategy and images demonstrating effective automated identification of cells can be seen in S7 and S8 Figs.

### Stochastic simulation of induction

The stochastic mathematical model was developed using the Hybrid Jump/Continuous Markov Stochastic Simulator (HyJCMSS), part of the Hybrid Stochastic Simulation for Supercomputers (Hy3S) suite [29]. The model consisted of 15 species and 38 biochemical reactions involved in the induction of expression of Q_L_ (S2 Table). The cell volume was assumed to be 10^−15^ L and was modeled to increase exponentially until cell division that occurred every 40 ± 4 minutes. Upon cell division, the number of proteins and mRNA molecules was halved. Initial conditions for all the species were obtained by solving equations S1-S15 (S1 Text) for steady-state when the extracellular concentration of cCF10 (***C***) was set to 0 ng ml^-1^ ***C***. For each simulation, 10,000 trials (corresponding to 10,000 cells) were carried out.

## Supporting information

S1 Fig Model for the mechanism of pCF10 induction resulting in Q_L_ transcription. Recipient cells produce lipoproteins (Pre-***C***) which are processed and exported from the cell (***C***_ex_). ***C***_ex_ is imported (***C***) into potential donor cells where ***C*** can interact with PrgX (*X*) to form *X*_*4*_*C*_*4*_ complexes. *X*_*4*_*C*_*4*_ complexes allow induced transcription of Q_L_ (which encodes the downstream conjugation genes) from the P_Q_ promoter. The ***I*** inhibitory peptide is produced from Pre-***I*** upon export. ***I***_ex_ is imported (***I***) where it can interact with *X* to form *X*_*4*_*I*_*4*_ complexes and prevent induction of Q_L_.
(TIFF)

S2 Fig *E*. *faecalis* HCR labeling protocol overview. (TIF)

S3 Fig Majority of cells are shown to express *ptsI* by HCR labeling indicating permeabilization effectiveness. Histograms show relative fluorescence intensity of HCR labeling for *ptsI* and *lacZ* or *prgB* transcripts. Scatter plots compare relative fluorescent intensity of *ptsI* and *lacZ* labeling or *ptsI* and *prgB* labeling within each cell. **(A)** JRC104+pBK2 samples. Left, 0 ng ml^-1^ at 0 minutes. Right, 5 ng ml^-1^ at 30 minutes. **(B)** OG1RF+pCF10 samples. Left 0 ng ml^-1^ at 0 minutes. Right, 5 ng ml^-1^ at 30 minutes.
(TIF)

S4 Fig The *lacZ* HCR signal is minimal in the absence of induction. Comparison of raw and Min/Max histogram stretched *lacZ* HCR signal with and without ***C*** addition. Cell envelope labeled with AF647: WGA shown for reference.
(TIFF)

S5 Fig Image analysis scheme for images of HCR labeled cells. (TIFF)

S6 Fig Image analysis sample output confirms identification of individual cells for analysis of HCR signal. Sample image of JRC104+pBK2 cells exposed to 10 ng ml^-1^ ***C***, 30 minutes after ***C*** addition. **(A)** Hoechst image after background subtraction and maximum intensity projection. **(B)** Binarized image resulting from Otsu’s thresholding method. **(C)** Objects identified by Regionprops function are defined by red dots. **(D)** Objects remaining after filtering based on size are defined by red dots. These objects were analyzed for HCR signal.
(TIFF)

S7 Fig Image analysis scheme for GFP images. (TIF)

S8 Fig Image analysis sample output confirms identification of individual cells for analysis of GFP signal. **(A)** Hoechst image after background subtraction and maximum intensity projection. **(B)** Binarized image. **(C)** Cell marker locations after distance transform and maximum detection. **(D)** Watershed transform on binarized image using cell marker locations. **(E)** Identified cells using SelectComponents and ComponentMeasurements are defined here by white circles. **(F)** Identified cells which were tracked throughout all 12 time points using ImageFeatureTrack are indicated here by white circles. Scale bar = 20 μm **(A**-**F)**.
(TIFF)

S9 Fig Measurement of induction response by flow cytometry analysis of HCR labeling shows similar induction dynamic as measurement by microscopic analysis. **(A)** Cells were gated by typical forward and side scatter (FSC and SSC) properties and Hoechst 33342 positive staining (405 nm) before analysis of Alexa Fluor 488 HCR *lacZ* staining. Shown here are the results of this gating for JRC104+pBK2 samples before and after treatment with 5 ng ml^-1^ ***C***. **(B)** Percent of cells with *lacZ* or *prgB* RNAs, expressed from pBK2 and pCF10 respectively, labeled by HCR and measured by flow cytometry over time after addition of varied concentrations of ***C***.
(TIF)

S1 Movie Time lapse of GFP fluorescence within cells with pCIE-GFP observed after *C* addition. (MOV)

S1 Table HCR probe sequences and amplifier fluorophore details. (PDF)

S2 Table List of reactions and parameter values used in the stochastic model. (PDF)

S3 Table Flow cytometry voltage settings and compensation matrix. (PDF)

S4 Table Strains and plasmids used in this study. (PDF)

S1 Text Updated mathematical model for pheromone induction. (PDF)

S1 Methods Flow cytometry analysis of HCR labeled cells. (PDF)
