# Supplementary figures and images for: Stochasticity in the enterococcal sex pheromone response revealed by quantitative analysis of transcription in single cells
Source: PLoS Genet. 2017 Jul 3;13(7):e1006878. doi: 10.1371/journal.pgen.1006878 (PMC5515443; doi:10.1371/journal.pgen.1006878)

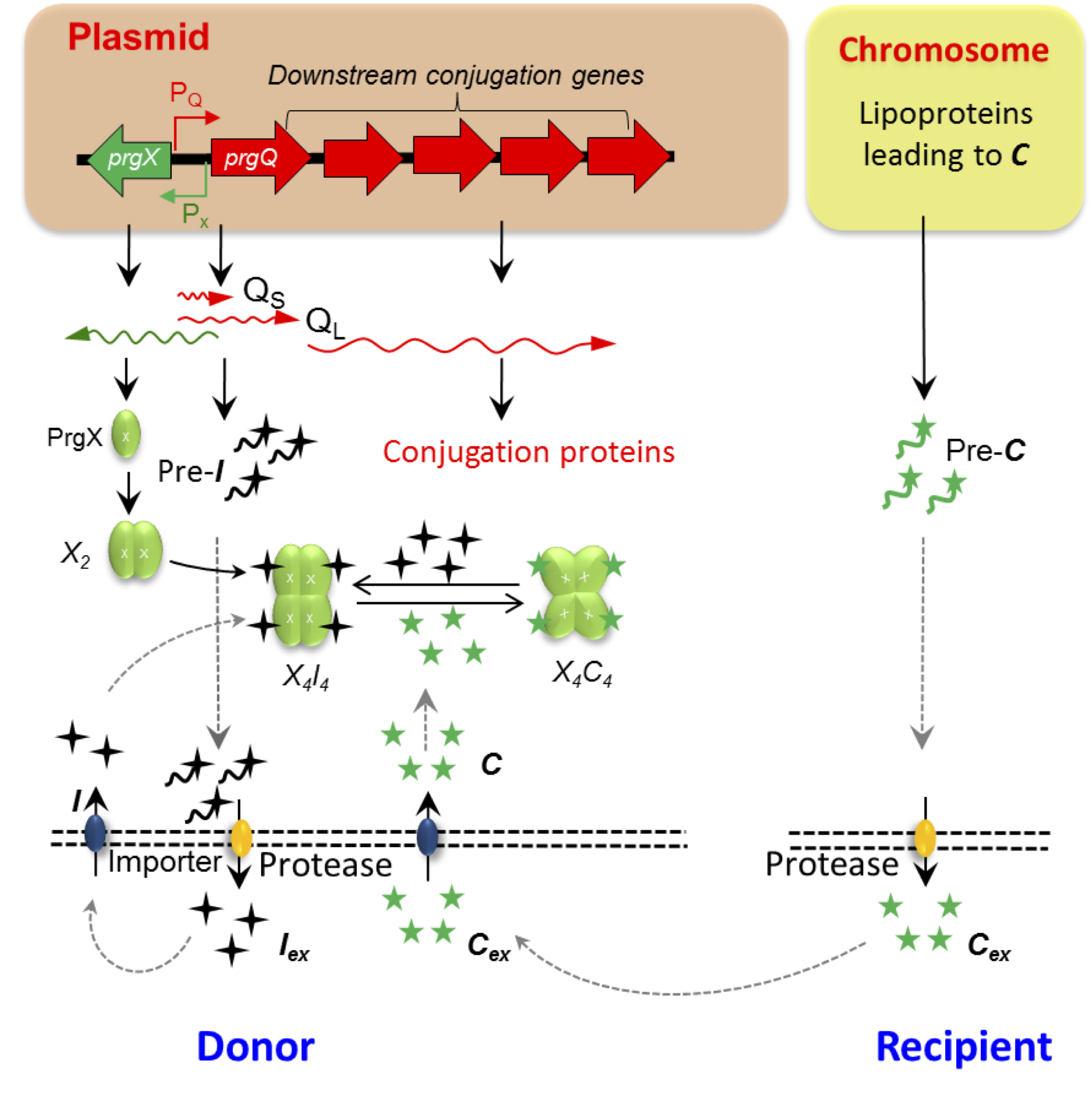

Supplement: S1 Fig — Recipient cells produce lipoproteins (Pre-C) which are processed and exported from the cell (Cex). Cex is imported (C) into potential donor cells where C can interact with PrgX (X) to form X4C4 complexes. X4C4 complexes allow induced transcription of QL (which encodes the downstream conjugation genes) from the PQ promoter. The I inhibitory peptide is produced from Pre-I upon export. Iex is imported (I) where it can interact with X to form X4I4 complexes and prevent induction of QL. (TIFF) [file pgen.1006878.s001.tiff]

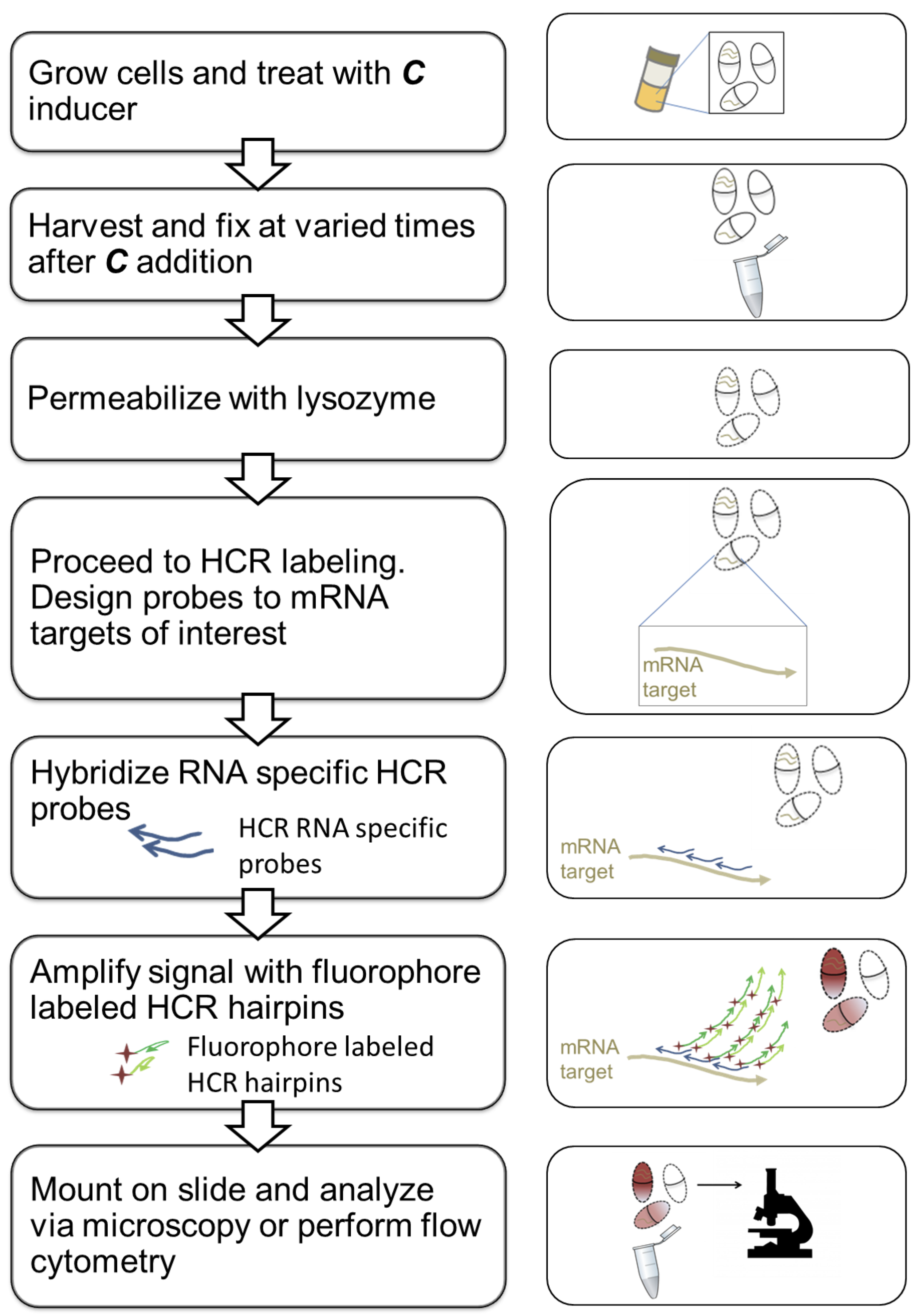

Supplement: S2 Fig — (TIF) [file pgen.1006878.s002.tif]

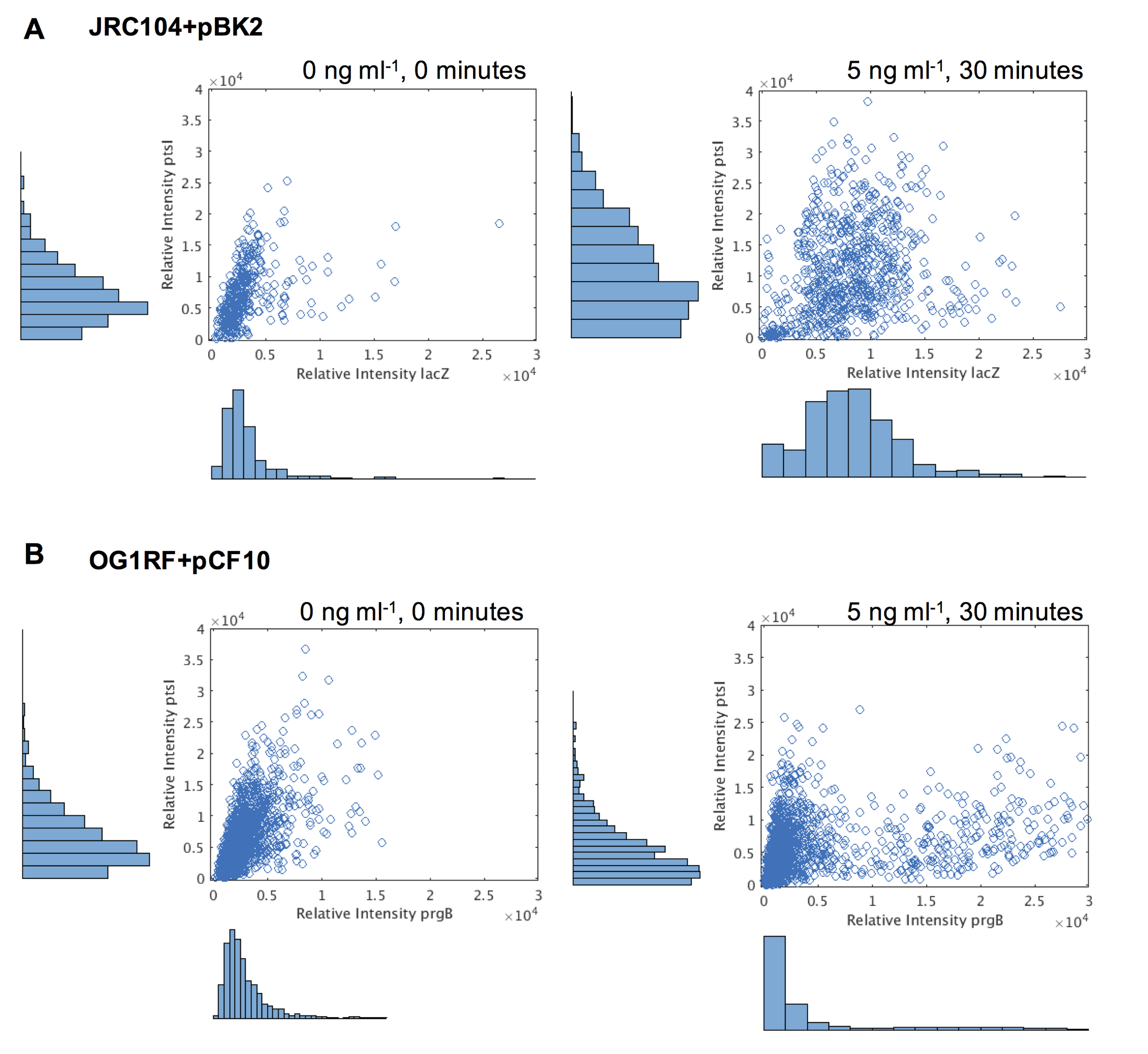

Supplement: S3 Fig — Histograms show relative fluorescence intensity of HCR labeling for ptsI and lacZ or prgB transcripts. Scatter plots compare relative fluorescent intensity of ptsI and lacZ labeling or ptsI and prgB labeling within each cell. (A) JRC104+pBK2 samples. Left, 0 ng ml-1 at 0 minutes. Right, 5 ng ml-1 at 30 minutes. (B) OG1RF+pCF10 samples. Left 0 ng ml-1 at 0 minutes. Right, 5 ng ml-1 at 30 minutes. (TIF) [file pgen.1006878.s003.tif]

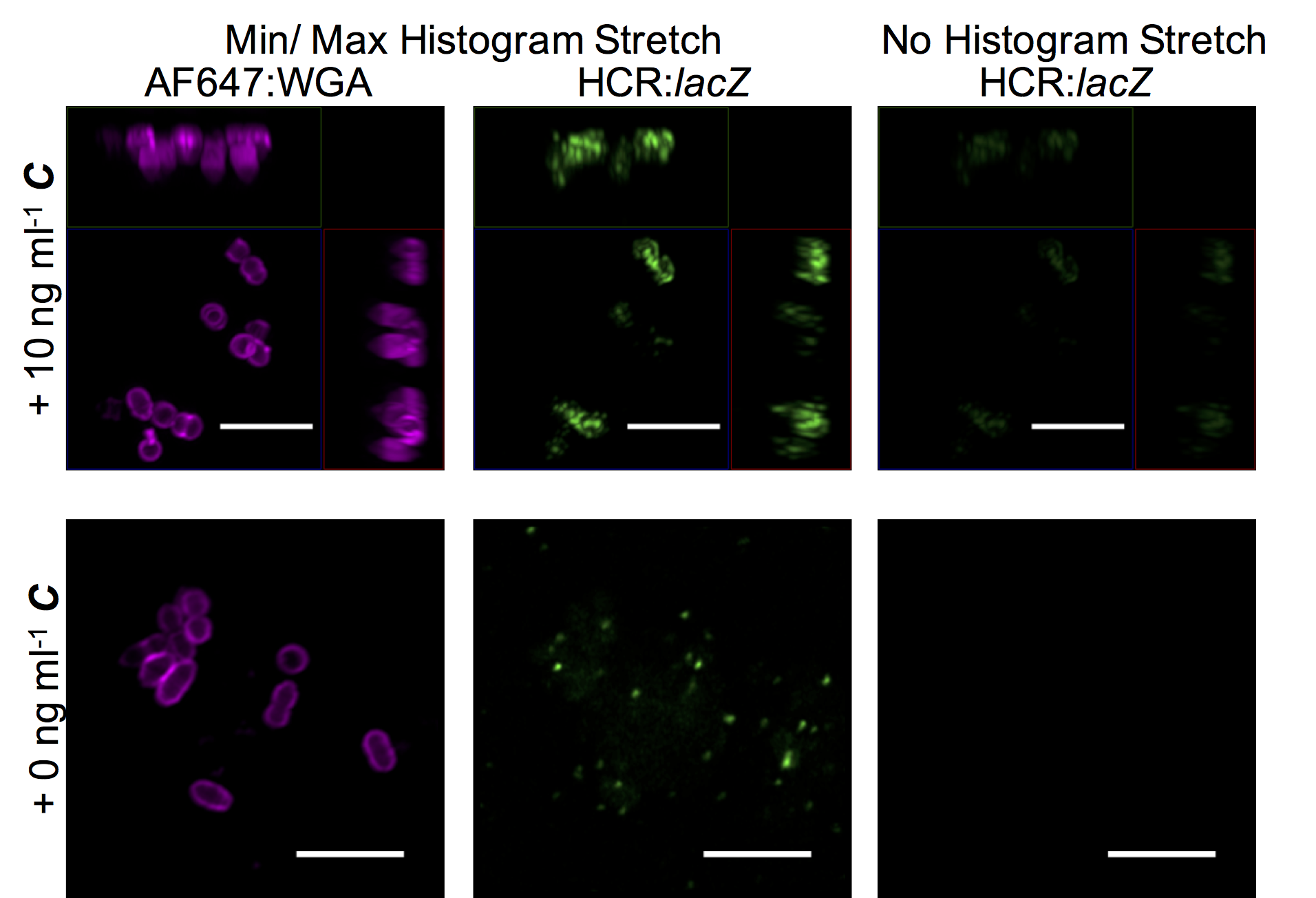

Supplement: S4 Fig — Comparison of raw and Min/Max histogram stretched lacZ HCR signal with and without C addition. Cell envelope labeled with AF647: WGA shown for reference. (TIFF) [file pgen.1006878.s004.tiff]

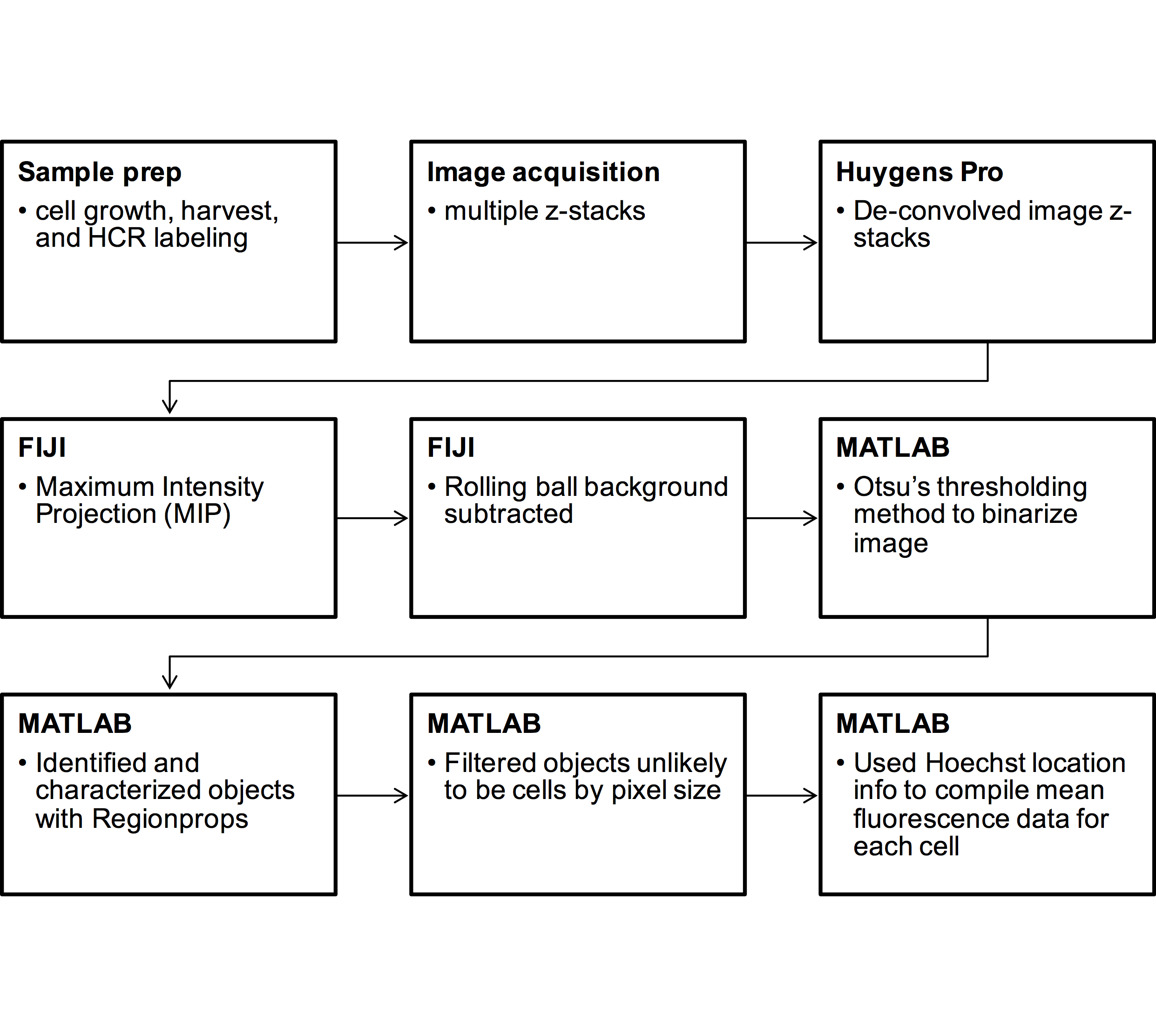

Supplement: S5 Fig — (TIFF) [file pgen.1006878.s005.tiff]

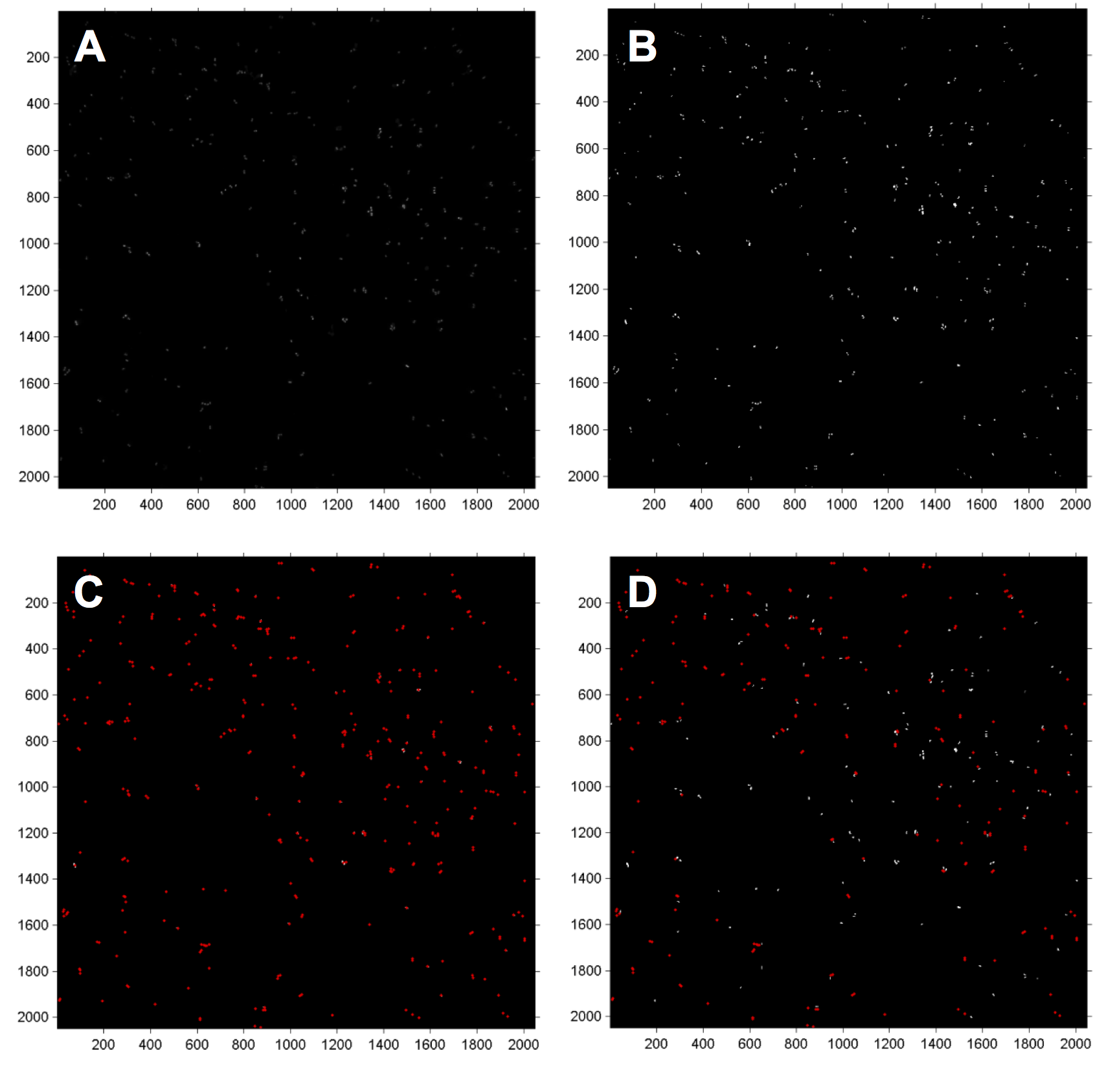

Supplement: S6 Fig — Sample image of JRC104+pBK2 cells exposed to 10 ng ml-1 C, 30 minutes after C addition. (A) Hoechst image after background subtraction and maximum intensity projection. (B) Binarized image resulting from Otsu’s thresholding method. (C) Objects identified by Regionprops function are defined by red dots. (D) Objects remaining after filtering based on size are defined by red dots. These objects were analyzed for HCR signal. (TIFF) [file pgen.1006878.s006.tiff]

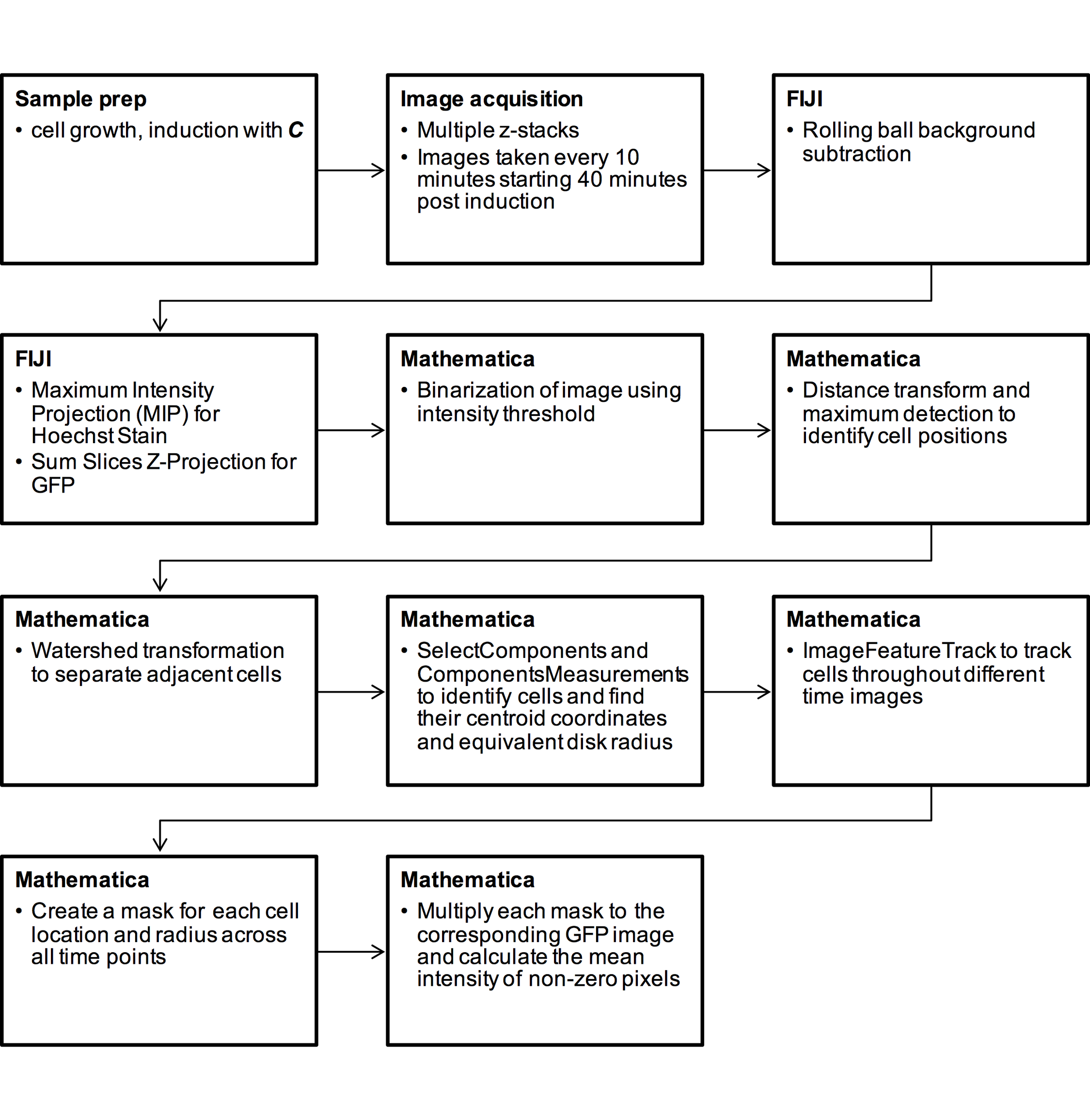

Supplement: S7 Fig — (TIF) [file pgen.1006878.s007.tif]

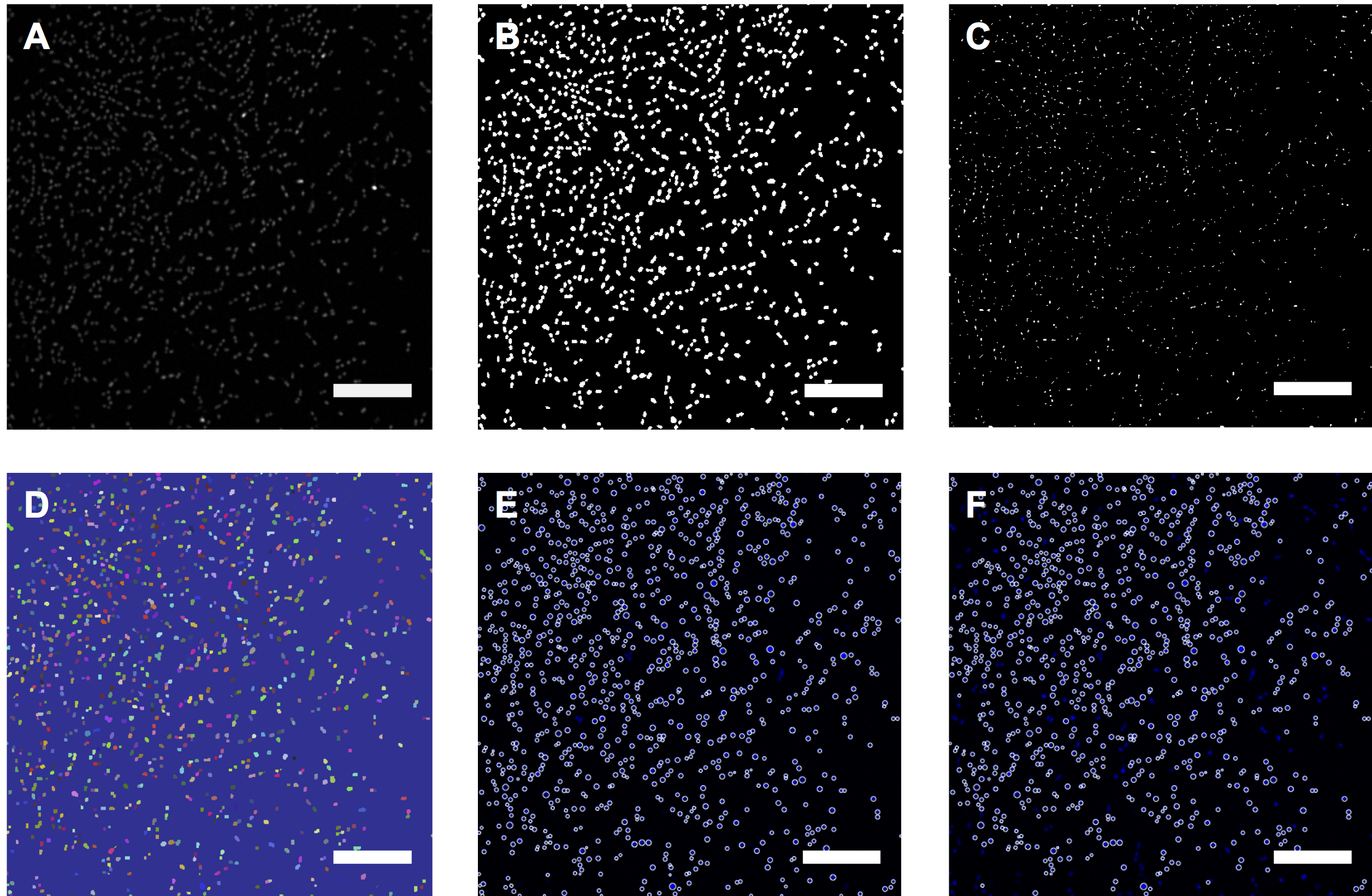

Supplement: S8 Fig — (A) Hoechst image after background subtraction and maximum intensity projection. (B) Binarized image. (C) Cell marker locations after distance transform and maximum detection. (D) Watershed transform on binarized image using cell marker locations. (E) Identified cells using SelectComponents and ComponentMeasurements are defined here by white circles. (F) Identified cells which were tracked throughout all 12 time points using ImageFeatureTrack are indicated here by white circles. Scale bar = 20 μm (A-F). (TIFF) [file pgen.1006878.s008.tiff]

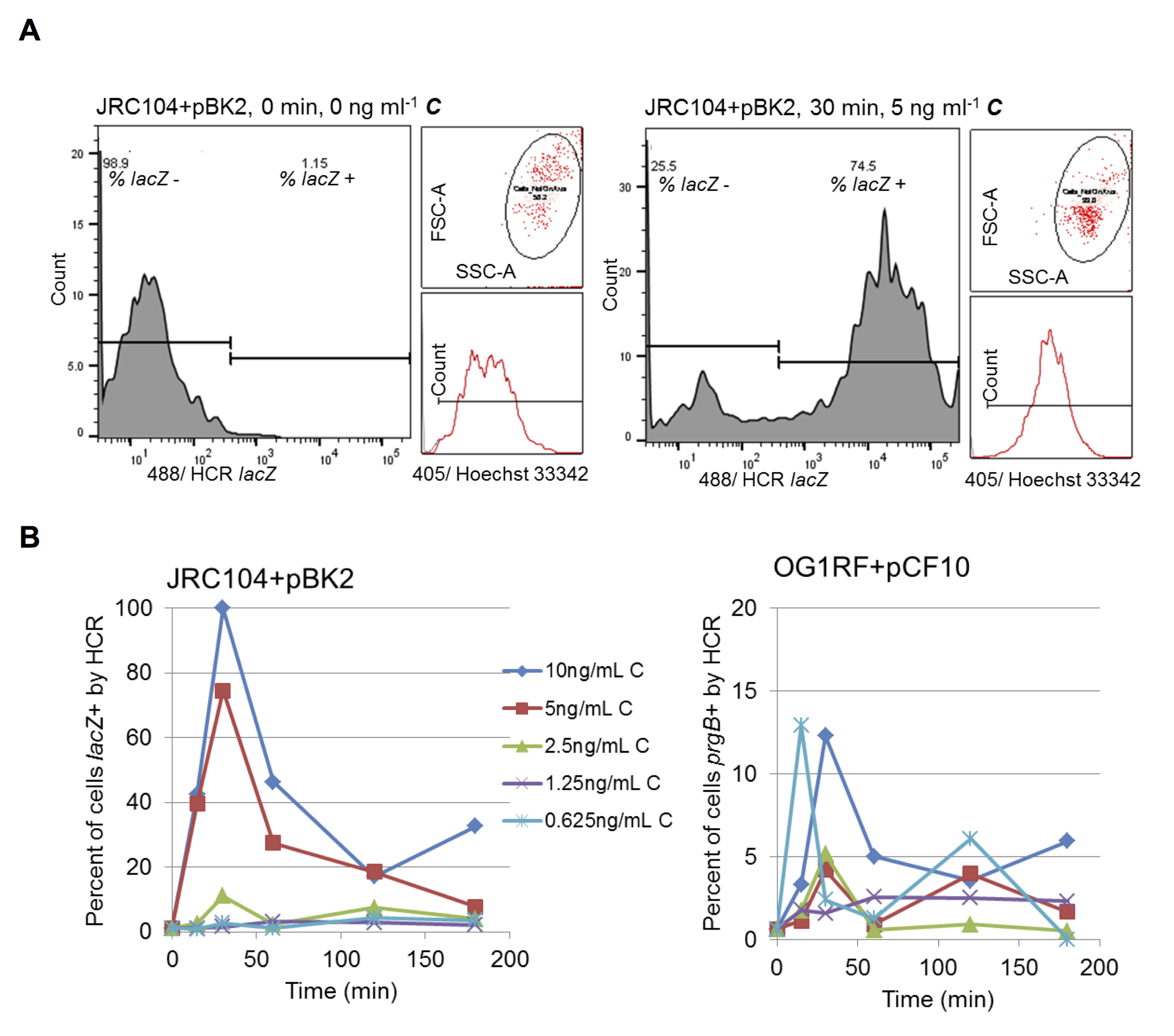

Supplement: S9 Fig — (A) Cells were gated by typical forward and side scatter (FSC and SSC) properties and Hoechst 33342 positive staining (405 nm) before analysis of Alexa Fluor 488 HCR lacZ staining. Shown here are the results of this gating for JRC104+pBK2 samples before and after treatment with 5 ng ml-1 C. (B) Percent of cells with lacZ or prgB RNAs, expressed from pBK2 and pCF10 respectively, labeled by HCR and measured by flow cytometry over time after addition of varied concentrations of C. (TIF) [file pgen.1006878.s009.tif]
