# Supplementary material for: Stochasticity in the enterococcal sex pheromone response revealed by quantitative analysis of transcription in single cells
Source: PLoS Genet. 2017 Jul 3;13(7):e1006878. doi: 10.1371/journal.pgen.1006878 (PMC5515443; doi:10.1371/journal.pgen.1006878)
Supplement: S1 Table — (PDF) [file pgen.1006878.s011.pdf]

HCR probes	Sequence (part of probe homologous to transcripts of interest)	Amplifier code
**ptsI-0**	CAGGTTGAACTAGCAGGTAAGCTTTAGCAACAGCAACGCCATCACTTGCG	B3
**ptsI-1**	GAGCATCGAAAACTTGCGCTTCTGCTTCACCAAGGCTTTGAGCTGCTTTG	
**ptsI-3**	GTGCTAAGATACGTTTGGCAACGTCACGAATATCTGCTGCACGCTCTTGC	
**ptsI-4**	GAAGTACGTCCGCCGATGTCTGTAACAAAGGCTTTAACATACGTACGGTC	
**ptsI-6**	GGTGTACCAATGTTTGCAGCTAACTCAACATGTTTGCCGTCAGCTGTCAC	
**ptsI-9**	TCTGTACGATATAAACCAACAGCTTCGCCGCCGTTTTTGTGTACGCCTTC	
**Elac-1**	CGACCTGCGTTTCACCCTGCCATAAAGAAACTGTTACCCGTAGGTAGTCA	B1
**Elac-4**	GGATGGTTCGGATAATGCGAACAGCGCACGGCGTTAAAGTTGTTCTGCTT	
**Elac-7**	TAAACGGGGATACTGACGAAACGCCTGCCAGTATTTAGCGAAACCGCCAA	
**Elac-11**	TCTCCGGCTGCGGTAGTTCAGGCAGTTCAATCAACTGTTTACCTTGTGGA	
**Elac-14**	TCCGGTAGGTTTTCCGGCTGATAAATAAGGTTTTCCCCTGATGCTGCCAC	
**Elac-18**	GCCACCAATCCCCATATGGAAACCGTCGATATTCAGCCATGTGCCTTCTT	
**prgB-3**	TTCGCTTCATTTTCAGCTGGTGCAACTTCTGTTGGCTGCCCTAGAGGTTC	B2D1
**prgB-5**	TTGAACGCATTGTTGGCCCCAAAATCAGAAACAAATTCCGCATGGCCACC	
**prgB-8**	CCCCATTGCTAAAACAACAGAGCCGTCCGCGTTGGTTTTCATCATTGCTG	
**prgB-16**	TACGCTAGAATAAAGGCTTGTGGGTCTTTGGCAGAAATCGTCACCGTGCC	
**prgB-22**	TGGCTCTACTGGTGCTTGCGGTGTTTTTGGTGTTGGTGGTACAATCACTG	

Transcript labeled	Amplifiers/ Fluorophore
**ptsI**	B3H1, B3H2 Alexa Fluor 546
**lacZ**	B1H1, B1H2 Alexa Fluor 546
**lacZ**	B1H1, B1H2 Alexa Fluor 488
**prgB**	B2H1, B2H2 Alexa Fluor 488
