# Supplementary material for: Stochasticity in the enterococcal sex pheromone response revealed by quantitative analysis of transcription in single cells
Source: PLoS Genet. 2017 Jul 3;13(7):e1006878. doi: 10.1371/journal.pgen.1006878 (PMC5515443; doi:10.1371/journal.pgen.1006878)
Supplement: S2 Table — (PDF) [file pgen.1006878.s012.pdf]

	Reactions	Parameter	Description	Value
1	$2X_2 + 4I \xrightarrow{k_{X-I,\,bind}} X_4I_4$	$k_{X-I,\,bind}$	Rate constant for ***I*** bind to ***X*₂** forming PrgX-I tetramers	8.01×10^{7} $M^{-1}s^{-1}$
2	$X_4I_4 \xrightarrow{k_{X-I,\,\mathrm{diss}}} 2X_2 + 4I$	$k_{X-C,\,diss}$	Rate constant for dissociation of PrgX-I tetramers into ***I*** and ***X*₂**	2.1×10^{-5} s^{-1}
3	$2X_2 + 4C \xrightarrow{k_{X-C,\,bind}} X_4C_4$	$k_{X-C,\,bind}$	Rate constant for ***C*** bind to ***X*₂** forming PrgX-C tetramers	1.38×10^{7} $M^{-1}s^{-1}$
4	$X_4C_4 \xrightarrow{k_{X-C,\,\mathrm{diss}}} 2X_2 + 4C$	$k_{X-C,\,diss}$	Rate constant for dissociation of PrgX-C tetramers into ***C*** and ***X*₂**	5.5×10^{-5} s^{-1}
5	$O + 2X_2 \xrightarrow{k_{O-X_2,bind}} OX_4$	$k_{O-X_2,bind}$	Rate constant for ***X*₂** binding to DNA	1×10^{6} $M^{-1}s^{-1}$
6	$OX_4 \xrightarrow{k_{O-X_2,diss}} O + 2X_2$	$k_{O-X_2,diss}$	Rate constant for ***X*₂** dissociating from DNA	1×10^{-2} s^{-1}
7	$O + X_4I_4 \xrightarrow{k_{O-X_4I_4,bind}} OX_4I_4$	$k_{O-X_4I_4,bind}$	Rate constant for $\boldsymbol{X_4I_4}$ binding to DNA	1×10^{8} $M^{-1}s^{-1}$
8	$OX_4I_4 \xrightarrow{k_{O-X_4I_4,diss}} O + X_4I_4$	$k_{O-X_4I_4,diss}$	Rate constant for $\boldsymbol{X_4I_4}$ dissociating from DNA	1×10^{-3} s^{-1}
9	$O + X_4C_4 \xrightarrow{k_{O-X_4C_4,bind}} OX_4C_4$	$k_{O-X_4C_4,bind}$	Rate constant for $\boldsymbol{X_4C_4}$ binding to DNA	1×10^{8} $M^{-1}s^{-1}$
10	$OX_4C_4 \xrightarrow{k_{O-X_4C_4,diss}} O + X_4C_4$	$k_{O-X_4C_4,diss}$	Rate constant for $\boldsymbol{X_4C_4}$ dissociating from DNA	1×10^{-3} s^{-1}
11	$O \xrightarrow{k_{P_Q,induced}} O + Q_{pre}$	$k_{P_Q,induced}$	Transcription rate of Pre-Q RNA in induced state	0.1 s^{-1}
12	$OX_4C_4 \xrightarrow{k_{P_Q,induced}} OX_4C_4 + Q_{pre}$	$k_{P_Q,induced}$	Transcription rate of Pre-Q RNA in induced state	0.1 s^{-1}
13	$OX_4 \xrightarrow{k_{P_Q,repressed}} OX_4 + Q_{pre}$	$k_{P_Q,\mathrm{repressed}}$	Transcription rate of Pre-Q RNA in repressed state	7.23×10^{-4} s^{-1}
14	$OX_4I_4 \xrightarrow{k_{P_Q,repressed}} OX_4I_4 + Q_{pre}$	$k_{P_Q,\mathrm{repressed}}$	Transcription rate of Pre-Q RNA in repressed state	7.23×10^{-4} s^{-1}
15	$O \xrightarrow{k_{Q_a,induced}} O + Q_a$	$k_{Q_a,induced}$	Transcription rate of anti-Q RNA in induced state	1.21×10^{-3} s^{-1}
16	$OX_4C_4 \xrightarrow{k_{Q_a,induced}} OX_4C_4 + Q_a$	$k_{Q_a,induced}$	Transcription rate of anti-Q RNA in induced state	1.21×10^{-3} s^{-1}
17	$OX_4 \xrightarrow{k_{Q_a,repressed}} OX_4 + Q_a$	$k_{Q_a,\mathrm{repressed}}$	Transcription rate of anti-Q RNA in repressed state	8.23×10^{-3} s^{-1}
18	$OX_4I_4 \xrightarrow{k_{Q_a,repressed}} OX_4I_4 + Q_a$	$k_{Q_a,\mathrm{repressed}}$	Transcription rate of anti-Q RNA in repressed state	8.23×10^{-3} s^{-1}

	Reactions	Parameter	Description	Value
19	$Q_{pre} \xrightarrow{k_{Q_L}} Q_L$	k_{Q_L}	Rate constant for Q_L synthesis from Q_{pre}	1 s^{-1}
20	$Q_{pre} + Q_a \xrightarrow{k_{Q_{pre}-Q_a}} Q_s$	$k_{Q_{pre}-Q_a}$	Rate constant of interaction between Q_{pre} and Q_a RNA	4.43×10^{8} $M^{-1}s^{-1}$
21	$Q_s \xrightarrow{k_{I_{ex}}} I_{ex}$	$k_{I_{ex}}$	Generation rate of extracellular iCF10	5×10^{-4} s^{-1}
22	$Q_L \xrightarrow{k_{I_{ex}}} I_{ex}$	$k_{I_{ex}}$	Generation rate of extracellular iCF10	5×10^{-4} s^{-1}
23	$I_{ex} \xrightarrow{k_{T_i}} I$	k_{T_i}	Transport rate constant of iCF10	1×10^{-3} s^{-1}
24	$\varnothing \xrightarrow{k_{Tc}} C$	k_{T_c}	Transport rate constant of cCF10	$1\times10^{-3}\times C_{ex}$ s^{-1}
25	$O \xrightarrow{k_{P_X,induced}} O + prgX$	$k_{P_X,induced}$	Transcription rate of *prgX* in induced state	1.21×10^{-5} s^{-1}
26	$OX_4C_4 \xrightarrow{k_{P_X,induced}} OX_4C_4 + prgX$	$k_{P_X,induced}$	Transcription rate of *prgX* in induced state	1.21×10^{-5} s^{-1}
27	$OX_4 \xrightarrow{k_{P_X,repressed}} OX_4 + prgX$	$k_{P_X,\mathrm{repressed}}$	Transcription rate of *prgX* in repressed state	1.02×10^{-2} s^{-1}
28	$OX_4I_4 \xrightarrow{k_{P_X,repressed}} OX_4I_4 + prgX$	$k_{P_X,\mathrm{repressed}}$	Transcription rate of *prgX* in repressed state	1.02×10^{-2} s^{-1}
29	$prgX \xrightarrow{k_{X,trans}} prgX + X_2$	$k_{X,trans}$	Rate constant for translation and subsequent dimerization of PrgX	2×10^{-3} s^{-1}
30	$X_2 \xrightarrow{\lambda_{X_2}} \varnothing$	λ_{X_2}	Degradation rate of apo-PrgX dimer	1×10^{-5} s^{-1}
31	$X_4C_4 \xrightarrow{\lambda_{X_4C_4}} \varnothing$	$\lambda_{X_4C_4}$	Degradation rate of PrgX-C tetramers	1×10^{-5} s^{-1}
32	$X_4I_4 \xrightarrow{\lambda_{X_4I_4}} \varnothing$	$\lambda_{X_4I_4}$	Degradation rate of PrgX-I tetramers	1×10^{-5} s^{-1}
33	$I \xrightarrow{\lambda_I} \varnothing$	λ_I	Degradation rate of intracellular iCF10	1×10^{-5} s^{-1}
34	$C \xrightarrow{\lambda_C} \varnothing$	λ_C	Degradation rate of intracellular cCF10	1×10^{-5} s^{-1}
35	$Q_L \xrightarrow{\lambda_{Q_L}} \varnothing$	λ_{Q_s}	Degradation rate of Q_L RNA	1×10^{-3} s^{-1}
36	$Q_s \xrightarrow{\lambda_{Q_s}} \varnothing$	λ_{Q_s}	Degradation rate of Q_s RNA	5×10^{-3} s^{-1}
37	$Q_a \xrightarrow{\lambda_{Qa}} \varnothing$	λ_{Q_a}	Degradation rate of Anti-Q RNA	1×10^{-3} s^{-1}
38	$prgX \xrightarrow{\lambda_{prgX}} \varnothing$	λ_{prgX}	Degradation rate of *prgX* RNA	2×10^{-4} s^{-1}
