# Supplementary material for: Stochasticity in the enterococcal sex pheromone response revealed by quantitative analysis of transcription in single cells
Source: PLoS Genet. 2017 Jul 3;13(7):e1006878. doi: 10.1371/journal.pgen.1006878 (PMC5515443; doi:10.1371/journal.pgen.1006878)
Supplement: S3 Table — (PDF) [file pgen.1006878.s013.pdf]

Flow Cytometry Voltage Settings

**Flow Cytometry Parameter**	**Used to characterize**	**Voltage**
FSC-A	Size	444
SSC-A	Granularity	187
PE-Texas Red-A	Alexa Fluor 546 HCR labeled *ptsI*	572
Alexa Fluor 488-A	Alexa Fluor 488 HCR labeled *lacZ* or *prgB*	352
Alexa Fluor 405-A	Hoechst 33342	429

Flow Cytometry Compensation Matrix

	**Alexa Fluor 488-A**	**Alexa Fluor 647-A**	**PE-Texas Red-A**	**Alexa Fluor 405-A**
**Alexa Fluor 488-A**	100	0	0.072	0.514
**Alexa Fluor 647-A**	0.056	100	0.192	0.197
**PE-Texas Red-A**	0.05	0.028	100	0
**Alexa Fluor 405-A**	0	0	0	100
