# Supplementary material for: Stochasticity in the enterococcal sex pheromone response revealed by quantitative analysis of transcription in single cells
Source: PLoS Genet. 2017 Jul 3;13(7):e1006878. doi: 10.1371/journal.pgen.1006878 (PMC5515443; doi:10.1371/journal.pgen.1006878)
Supplement: S4 Table — (PDF) [file pgen.1006878.s014.pdf]

Strains	Description	Reference
OG1	Reference strain	33
OG1RF	OG1 derivative with Rifampicin and Fusidic acid resistance	34, 36
OG1Sp	OG1 derivative with Spectinomycin resistance	37
JRC104	OG1RF derivative that does not produce the cCF10 peptide due to a nonsense point mutation in the cCF10 encoding *ccfA* gene	37

Plasmids	Description	Reference
pBK2	Contains pCF10 regulatory region, has inducible expression of *lacZ* in lieu of genes encoding conjugation machinery, encodes for Chloramphenicol resistance	38
pCF10	Native *Enterococcus faecalis* conjugative plasmid encoding for Tetracycline resistance and inducible expression of genes for conjugation machinery	34, 35
pCIE-GFP	Derivative of the pBK2, contains pCF10 regulatory region, has inducible expression of gfp in lieu of genes encoding conjugation machinery, encodes for Chloramphenicol resistance	37
