# Supplementary material for: Stochasticity in the enterococcal sex pheromone response revealed by quantitative analysis of transcription in single cells
Source: PLoS Genet. 2017 Jul 3;13(7):e1006878. doi: 10.1371/journal.pgen.1006878 (PMC5515443; doi:10.1371/journal.pgen.1006878)
Supplement: S1 Text — (PDF) [file pgen.1006878.s015.pdf]

Modelling the *prgX-prgQ* gene network

A mathematical model for pheromone induction including the intracellular molecular events (i.e. interaction of signaling peptides, multiple transcriptional and post-transcriptional regulations), was described previously [18,19]. We recently gained more knowledge on the oligomeric states of apo-PrgX and PrgX/peptide complexes and measured the binding constants of the peptides to apo-PrgX and PrgX complexes to the DNA [28]. We further determined some kinetic constants using RNA-Seq.

The measured binding constants of both the peptides to PrgX are very high and are similar in value. Peptides bound to PrgX remains bound until degradation. Both the peptides bind to apo-PrgX and do not compete with each other for pre-formed PrgX-peptide complexes. In order to facilitate this condition, the intracellular concentrations of apo-PrgX dimers ($\boldsymbol{X_2}$) is likely to be in large excess. The induction and shut down of the P_Q promoter is controlled by the competition of $\boldsymbol{X_2}$, $\boldsymbol{X_4C_4}$, and $\boldsymbol{X_4I_4}$ for the operator site. An overview of the mechanism of pCF10 induction utilized by the new stochastic model is illustrated in S1 Fig.

Peptides binding to PrgX

$$2X_2 + 4C \xrightleftharpoons{K_{X-C}} X_4C_4$$

$$2X_2 + 4I \xrightleftharpoons{K_{X-I}} X_4I_4$$

$$\frac{K_{X-C}}{K_{X-I}} \approx 1$$

PrgX complexes binding to operator region of the DNA

$$O + X_4C_4 \xrightleftharpoons{K_{O-X_4C_4}} OX_4C_4$$

$$O + X_4I_4 \xrightleftharpoons{K_{O-X_4I_4}} OX_4I_4$$

$$O + 2X_2 \xrightleftharpoons{K_{O-X_2}} OX_4$$

$$K_{O-X_4C_4} \approx K_{O-X_4I_4}$$

PrgX by itself can bind to DNA and cause a suppressive effect. Though apo-PrgX binds to DNA (***O***) with a lower affinity, high intracellular concentration allows it to bind to DNA, forming (***OX_4***), suppressing the P_Q promoter under basal uninduced conditions.

$$[N] \approx [OX_4] + [OX_4C_4] + [OX_4I_4]$$

Control of induction of the P_Q promoter and an order of magnitude estimate of the intracellular concentrations of various species under various stages of induction are shown below.

	X_2	X_4C_4	X_4I_4	O	OX_4	OX_4C_4	OX_4I_4
Uninduced state	100-1000	0	1-5	0	3	0	2
Induced state	100-1000	1-5	5-10	0	1	1-2	2-3
Post-induced state	100-1000	1-5	20-50	0	1	0	4

Under basal uninduced conditions, the P_Q promoter is suppressed by apo-PrgX and PrgX-iCF10 tetramers in ***OX_4*** and ***OX_4I_4*** states. When the donor cells are exposed to ***C***, extracellular ***C*** in imported in which then binds to apo-PrgX dimers (***X_2***) forming ***X_4C_4***. ***X_4C_4*** having two orders of magnitude higher affinity for DNA replaces apo-PrgX on the plasmids, inducing the P_Q promoter. The induction of just one plasmid may be sufficient to generate enough ***Q_L*** transcripts to allow synthesis of the conjugation machinery. Induction of P_Q promoter increases the synthesis of ***I*** which is processed and secreted out. Extracellular ***I*** (***I_{ex}***), is then re-imported back in by the donor cells where it binds to apo-PrgX dimers forming ***X_4I_4***. High-performance liquid chromatography fractionation of supernatant of pCF10 carrying *E. faecalis* cultures shows that ***I*** and ***C*** are secreted typically in a molar ratio of 10–100 to 1 [12]. Both ***X_4C_4*** and ***X_4I_4*** have similar

binding affinities towards DNA. However due to higher concentration of $\boldsymbol{X_4I_4}$, it preferably binds to the DNA causing a suppressive effect and shutting down the synthesis of conjugation machinery.

The binding events are summarized in the reactions below:

$$2X_2 + 4C \underset{k_{X-C,\, diss}}{\overset{k_{X-C,\, bind}}{\rightleftharpoons}} X_4C_4 \tag{1}$$

$$2X_2 + 4I \underset{k_{X-I,\, diss}}{\overset{k_{X-I,\, bind}}{\rightleftharpoons}} X_4I_4 \tag{2}$$

$$O + 2X_2 \underset{k_{\text{O-X}_4,\, diss}}{\overset{k_{\text{O-X}_4,\, bind}}{\rightleftharpoons}} OX_4 \tag{3}$$

$$O + X_4C_4 \underset{k_{\text{O-X}_4\text{C}_4,\, diss}}{\overset{k_{\text{O-X}_4\text{C}_4,\, bind}}{\rightleftharpoons}} OX_4C_4 \tag{4}$$

$$O + X_4I_4 \underset{k_{\text{O-X}_4 I_4,\, diss}}{\overset{k_{\text{O-X}_4 I_4,\, bind}}{\rightleftharpoons}} OX_4I_4 \tag{5}$$

A set of mass action differential equations describing the balance of various transcripts, signaling molecules ***C*** and ***I*** involved in the *prgQ-prgX* genetic switch is provided below.

$$\frac{d[OX_4]}{dt} = k_{O-X_2,\, bind}[O][X_2] - k_{O-X_2,\, diss}[OX_4] \tag{S1}$$

$$\frac{d[OX_4C_4]}{dt} = k_{O-X_4C_4,\, bind}[O][X_4C_4] - k_{O-X_4C_4,\, diss}[OX_4C_4] \tag{S2}$$

$$\frac{d[OX_4I_4]}{dt} = k_{O-X_4I_4,\, bind}[O][X_4I_4] - k_{O-X_4I_4,\, diss}[OX_4I_4] \tag{S3}$$

$$\frac{d[O]}{dt} = -\frac{d[OX_4]}{dt} - \frac{d[OX_4I_4]}{dt} - \frac{d[OX_4I_4]}{dt} \tag{S4}$$

Transcription from both P_Q and P_X depends on the state of the pCF10 DNA. Equations S1-S4 describe the balance of the various states of the pCF10 DNA i.e. DNA bound to $\boldsymbol{X_4}$ ($\boldsymbol{OX_4}$), $\boldsymbol{X_4C_4}$ ($\boldsymbol{OX_4C_4}$), $\boldsymbol{X_4I_4}$ ($\boldsymbol{OX_4I_4}$) and free DNA ($\boldsymbol{O}$). Since the apo-PrgX dimer ($\boldsymbol{X_2}$) concentration is significantly higher when compared to DNA ($\boldsymbol{O}$), $\boldsymbol{X_2}$ concentration effectively remains constant during the reaction because its consumption would be so small that the change in concentration becomes negligible. Hence, we assume the binding of $\boldsymbol{X_2}$ to DNA in reaction 3 follows first order kinetics with respect to both $\boldsymbol{X_2}$ and $\boldsymbol{O}$ as described in Equation S1. Equations S2 and S3 mathematically describe the species balances for $\boldsymbol{OX_4C_4}$ and $\boldsymbol{OX_4I_4}$ based on reactions 4 and 5. The binding constants for $\boldsymbol{X_4}$, $\boldsymbol{X_4C_4}$, and $\boldsymbol{X_4I_4}$ to DNA ($\boldsymbol{O}$) were estimated using EMSAs [28]. The forward and backward reaction rates were assumed to be in the order of typical DNA binding reactions. Equation S5 describes the balance on free DNA ($\boldsymbol{O}$) under the assumption that the total plasmid copy number remains constant.

$$\frac{d\left[Q_{pre}\right]}{dt} = k_{P_Q,induced}\left(\left[O\right]+\left[OX_4C_4\right]\right)+k_{P_Q,\text{repressed}}\left(\left[OX_4\right]+\left[OX_4I_4\right]\right)-k_{Q_L}\left[Q_{pre}\right]-k_{Q_{pre}-Q_a}\left[Q_{pre}\right]\left[Q_a\right] \tag{S5}$$

$$\frac{d\left[Q_L\right]}{dt} = k_{Q_L}\left[Q_{pre}\right]-\left(\lambda_{Q_L}+\mu\right)\left[Q_L\right] \tag{S6}$$

$$\frac{d\left[Q_s\right]}{dt} = k_{Q_{pre}-Q_a}\left[Q_{pre}\right]\left[Q_a\right]-\left(\lambda_{Q_s}+\mu\right)\left[Q_s\right] \tag{S7}$$

Equation S5 describes the transcription from P_Q promoter, which depends on the state of DNA that may be under an induced state ($\boldsymbol{O}$, $\boldsymbol{OX_4C_4}$) or under repressed conditions ($\boldsymbol{OX_4}$, $\boldsymbol{OX_4I_4}$). The nascent transcript $\boldsymbol{Q_{pre}}$ either may be transcribed into full-length transcript $\boldsymbol{Q_L}$ or get truncated into shorter transcript $\boldsymbol{Q_S}$ due to antisense interaction with 104-nt Anti-Q RNA ($\boldsymbol{Q_a}$) as shown by the fourth term in Equation S5. The remaining $\boldsymbol{Q_{pre}}$ transcript continues to elongate as a first

order reaction with respect to $\boldsymbol{Q_{pre}}$. Since nascent transcript $\boldsymbol{Q_{pre}}$ is an intermediate species, no dilution or degradation term has been considered. Equations S6 and S7 describe the synthesis of $\boldsymbol{Q_L}$ and $\boldsymbol{Q_S}$ transcripts.

$$\frac{d[Q_a]}{dt} = k_{Q_a,induced}\left([O]+[OX_4c_4]\right)+k_{Q_a,\mathrm{repressed}}\left([OX_4]+[OX_4i_4]\right)-k_{Q_{pre}-Q_a}\left[Q_{pre}\right][Q_a]-(\lambda_{Q_a}+\mu)[Q_a] \quad \text{(S8)}$$

$$\frac{d[prgX]}{dt} = k_{P_X,induced}\left([O]+[OX_4c_4]\right)+k_{P_X,\mathrm{repressed}}\left([OX_4]+[OX_4i_4]\right)-\left(\lambda_{prgX}+\mu\right)[prgX] \quad \text{(S9)}$$

Equations S8 and S9 describe transcription from P_X promoter, which is involved in the synthesis of Anti-Q RNA ($\boldsymbol{Q_a}$) and full-length transcript for PrgX protein.

$$\begin{aligned}\frac{d[X_2]}{dt} = {} & k_{X,trans}[prgX]-2k_{X-C,\,bind}[X_2][C]+2k_{X-C,\,diss}[X_4C_4]-2k_{X-I,\,bind}[X_2][I] \\ & +2k_{X-I,\,diss}[X_4I_4]-2k_{O-X_2,\,bind}[O][X_2]+2k_{O-X_2,\,diss}[OX_4]-\left(\lambda_{X_2}+\mu\right)[X_2]\end{aligned} \quad \text{(S10)}$$

$$\begin{aligned}\frac{d[X_4C_4]}{dt} = {} & k_{X-C,\,bind}[X_2][C]-k_{X-C,\,diss}[X_4C_4]-k_{O-X_4C_4,\,bind}[O][X_4C_4] \\ & +k_{O-X_4C_4,\,diss}[OX_4C_4]-\left(\lambda_{X_4C_4}+\mu\right)[X_4C_4]\end{aligned} \quad \text{(S11)}$$

$$\begin{aligned}\frac{d[X_4I_4]}{dt} = {} & k_{X-I,\,bind}[X_2][I]-k_{X-I,\,diss}[X_4I_4]-k_{O-X_4C_4,\,bind}[O][X_4I_4] \\ & +k_{O-X_4I_4,\,diss}[OX_4I_4]-\left(\lambda_{X_4I_4}+\mu\right)[X_4I_4]\end{aligned} \quad \text{(S12)}$$

Equations S10-S12 describe the species balance of $\boldsymbol{X_2}$, $\boldsymbol{X_4C_4}$, and $\boldsymbol{X_4I_4}$ based on reactions 1, 2, 4 and 5. Translation of PrgX transcripts and subsequent dimerization were combined into a single step and described by kinetic constant $\boldsymbol{k_{X\,,trans}}$. The binding reactions of peptides to apo-PrgX were assumed to be first-order with respect to both apo-PrgX dimers ($\boldsymbol{X_2}$) and the respective peptides (***I*** and ***C***) and the rate constants were experimentally determined using SPR [28].

$$\frac{d[I_{ex}]}{dt} = k_{I_{ex}}\left(Q_L + Q_S\right) - k_{T_i}\left([I_{ex}] - [I]\right) \tag{S13}$$

$$\frac{d[C]}{dt} = k_{T_c}\left([C_{ex}] - [C]\right) - 4k_{X-C,\,bind}[X_2][c] + 4k_{X-C,\,diss}[X_4C_4] - \left(\lambda_C + \mu\right)[C] \tag{S14}$$

$$\frac{d[I]}{dt} = k_{T_i}\left([I_{ex}] - [I]\right) - 4k_{X-I,\,bind}[X_2][I] + 4k_{X-I,\,diss}[X_4I_4] - \left(\lambda_I + \mu\right)[I] \tag{S15}$$

The dynamics of extracellular iCF10 ($\boldsymbol{I_{ex}}$), intracellular iCF10 (***I***), extracellular cCF10 ($\boldsymbol{C_{ex}}$), and cCF10 (***C***) are shown in Equations S13-S15. iCF10 is synthesized by pCF10 under leader sequence of transcripts produced from the P_Q promoter and is part of both $\boldsymbol{Q_s}$ and $\boldsymbol{Q_L}$ transcripts. The peptide is translated as pre-iCF10, which is cleaved and processed into active iCF10 when exported from cells. Combining its translation and export, the rate of iCF10 synthesis is modeled to be proportional to the sum of $\boldsymbol{Q_s}$ and $\boldsymbol{Q_L}$. The transport of signaling molecules cCF10 and iCF10 across the membrane protein PrgZ is modeled as a first-order reaction dependent on the concentration difference between extracellular and intracellular concentrations of respective peptides.

Initial conditions for all the species were obtained by solving equations S1-S15 for steady-state when the extracellular concentration of cCF10 (***C***) was set to zero using function "ode15s" in Matlab (version 2014b; MathWorks). These initial conditions were used in the stochastic model. Subsequently, the extracellular concentration of cCF10 (***C***) was fixed at a desired value and the Hy3S based stochastic simulation was executed. A list of reactions and parameter value used in the stochastic model can be found in S2 Table.
