# Supplementary material for: Stochasticity in the enterococcal sex pheromone response revealed by quantitative analysis of transcription in single cells
Source: PLoS Genet. 2017 Jul 3;13(7):e1006878. doi: 10.1371/journal.pgen.1006878 (PMC5515443; doi:10.1371/journal.pgen.1006878)
Supplement: S1 Methods — (PDF) [file pgen.1006878.s016.pdf]

S1 Methods

Flow cytometry analysis of HCR labeled cells. 10 μl of remaining HCR labeled cell samples in suspension were diluted in 1 ml PBS and analyzed by flow cytometry to further support results obtained by microscopic image analysis. Cells were analyzed for fluorescence from Hoechst 33342, Alexa Fluor 488 corresponding to HCR labeled *lacZ* or *prgB*, and Alexa Fluor 546 corresponding to HCR labeled *ptsI* using a BD LSR Fortessa flow cytometer and BD FACSDiva Software (version 8.0). Data was analyzed using FlowJo software (version 10.2). Summarized results of flow cytometry analysis can be found in S9 Fig. Flow cytometry settings can be found in S3 Table.
